# Hybrid Event–Frame Sensing for Human-Perceptual Imaging and Machine Vision

**DOI:** 10.3390/s26165127

**Published:** 2026-08-13

**Authors:** Paul K. J. Park, Junseok Kim, Juhyun Ko

**Affiliations:** 1 Samsung Electronics, Hwaseong 18448, Gyeonggi-do, Republic of Korea; junseok7.kim@samsung.com (J.K.); juhyun03.ko@samsung.com (J.K.); 2 Department of Semiconductor Display, Gachon University, Seongnam 13120, Gyeonggi-do, Republic of Korea

**Keywords:** hybrid event–frame sensing, dynamic vision sensor, event vision sensor, CMOS image sensor, CIS–DVS integration, stacked image sensor, homogeneous-pixel hybrid sensing system, demosaicing, event–frame fusion, computational ISP

## Abstract

**Highlights:**

**What are the main findings?**

- This review presents a sensor-oriented taxonomy of hybrid event–frame sensing architectures and systems, including dual-camera event–frame systems, optically aligned event–frame systems, pixel-level shared hybrid image sensors, stacked CIS–DVS hybrid image sensors, homogeneous-pixel sensing systems, and event-only reconstruction systems.
- It analyzes key sensor specifications—latency, spatial resolution, color fidelity, power consumption, and form factor—and links them to configuration and design choices for hybrid event–frame sensing.

**What are the implications of the main findings?**

- Stacked CIS–DVS hybrid image sensors are identified as one of the most competitive architectures across the reviewed specifications, offering a strong balance among compact form factor, synchronized event–frame sensing, spatial resolution, power efficiency, and system integration.
- Color fidelity, demosaicing, event-pixel ratio, calibration, and edge-AI deployment remain key challenges for future human-perceptual imaging and machine-vision applications.

**Abstract:**

Frame-based RGB image sensors and event-based vision sensors provide complementary sensing capabilities for human-perceptual imaging and machine vision. RGB image sensors capture dense spatial, color, and texture information that is essential for human-viewable imaging, semantic recognition, and conventional image signal processing pipelines. In contrast, dynamic vision sensors (DVSs) and event vision sensors (EVSs) asynchronously detect local brightness changes and provide sparse temporal information with low latency, high temporal resolution, and reduced redundant data output. Because neither modality alone satisfies all requirements of emerging vision systems, hybrid event–frame sensing has become an important direction for compact, low-latency, and energy-efficient sensing. This review presents a sensor-oriented taxonomy of hybrid event–frame sensing architectures and systems, including dual-camera event–frame systems, optically aligned event–frame systems, pixel-level shared hybrid image sensors, stacked CIS–DVS hybrid image sensors, homogeneous-pixel sensing systems, and event-only reconstruction systems. We analyze key sensor specifications, including latency, spatial resolution, color fidelity, power consumption, and form factor, and discuss how these specifications guide sensor configuration and design. The review identifies stacked CIS–DVS sensors as one of the most balanced and competitive architectures because they can support compact integration, synchronized event–frame sensing, and on-chip processing. However, important challenges remain, including color fidelity, demosaicing, event-pixel ratio optimization, calibration, benchmarking, and edge-AI deployment. Finally, we emphasize that future hybrid event–frame sensing systems should be developed through sensor–algorithm–ISP–AI co-design. This review provides practical guidelines for developing next-generation hybrid event–frame sensing systems for both human-perceptual imaging and machine vision.

## 1. Introduction

Conventional frame-based RGB image sensors, especially CMOS active-pixel sensors (APSs), have enabled compact digital cameras, mobile imaging, machine vision, surveillance, robotics, and autonomous systems by providing dense spatial, color, and texture information in regularly sampled image frames [1,2,3]. Over the last three decades, continuous advances in pinned photodiodes, backside illumination, stacked image sensors, on-chip analog-to-digital conversion, image signal processors (ISPs), and computational photography have significantly improved resolution, sensitivity, dynamic range, noise performance, and form factor [1,2,3,4]. These developments have made frame-based RGB sensors the dominant visual front end for both human-viewable imaging and machine perception. Despite this success, frame-based sensing has intrinsic limitations. These limitations are especially important for emerging applications such as high-speed imaging, augmented and virtual reality, autonomous driving, mobile robotics, always-on AIoT sensing, and edge-AI systems, where low latency, low power, robustness, and compact form factor are simultaneously required.

**Figure 1:**
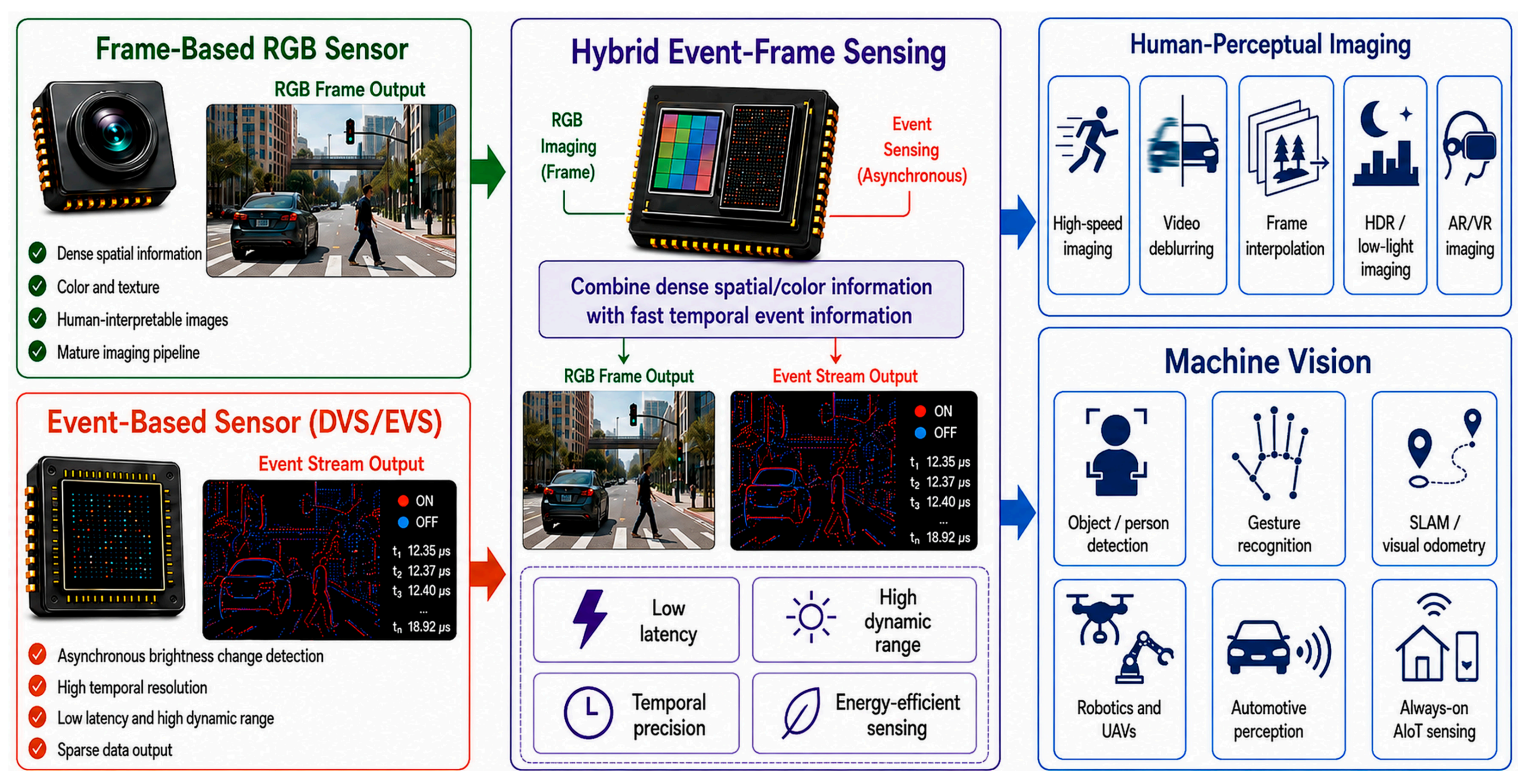
Concept of hybrid event–frame sensing for human-perceptual imaging and machine vision. Frame-based RGB sensors provide dense spatial, color, and texture information, whereas event-based DVS/EVS devices provide asynchronous brightness-change events with high temporal resolution, low latency, high dynamic range, and sparse data output. A hybrid event–frame system combines RGB frames and event streams to support both human-perceptual imaging and machine-vision applications. The optimal configuration requires application-specific co-design of the sensor architecture, fusion algorithm, and edge-AI processor. Original schematic created by the authors.

Event-based vision sensors, also known as dynamic vision sensors (DVSs) or event cameras, were developed to overcome some of these limitations by adopting an asynchronous sensing principle inspired by biological vision [5,6,7,8]. Instead of capturing full frames at fixed time intervals, a DVS pixel independently detects local brightness changes and generates an event when the logarithmic intensity change exceeds a predefined contrast threshold [5]. Because events are generated only when visual changes occur, event cameras provide sparse, low-latency, visual streams with high temporal resolution and dynamic range, and reduced redundant data output [5,8]. These characteristics make event-based sensing attractive for high-speed motion analysis, optical flow, visual odometry, SLAM, gesture recognition, robotics, automotive perception, and neuromorphic edge computing [8,9,10,11,12,13,14]. However, event-based sensors have their own limitations. Event streams are sensitive to bias settings, threshold mismatch, background activity, temporal noise, and illumination-dependent event generation [15,16,17]. Furthermore, many conventional computer-vision algorithms and datasets are built around frame-based images, making direct deployment of event-only systems difficult in existing machine-vision pipelines. As a result, event cameras and RGB frame cameras should not be regarded as competing technologies, but as complementary sensing modalities. Hybrid event–frame sensing has emerged from this complementarity. In a hybrid system, event-based sensing provides information on rapid temporal changes, motion cues, high dynamic range, and low-latency triggering, while frame-based RGB sensing provides dense spatial information, color, texture, and human-interpretable visual appearance. This combination can support both human-oriented imaging tasks, such as high-speed video reconstruction, video deblurring, frame interpolation, HDR imaging, and low-light imaging, and machine-oriented perception tasks, such as object detection, gesture recognition, SLAM, automotive perception, robotics, and always-on AIoT sensing, as shown in Figure 1 [8,9,10,11,12,13,14,18,19,20,21,22].

Several architectural approaches have been explored to combine event and frame sensing. The simplest is a loosely coupled dual-camera system, with an event camera and an RGB camera mounted together and calibrated geometrically and temporally. A second uses optically aligned event–frame systems, often with beam splitters or shared optical paths, to improve spatial alignment. A third is the pixel-level shared sensor, which integrates APS frame readout and DVS event readout within the same pixel array, with both functions sharing a common optical path, which gives intrinsic spatial alignment but increases pixel-circuit complexity and often limits color or pixel scaling. A fourth is stacked or co-integrated single-chip hybrid sensing, distributing frame and event functions across vertically integrated wafers or tightly coupled layers. A fifth is homogeneous-pixel hybrid sensing, where a conventional CIS array generates event-like pseudo-DVS information through high-speed temporal sampling. Finally, event-only reconstruction systems use pure DVS/EVS hardware and generate grayscale video algorithmically. Recent high-resolution RGB sensors with embedded event pixels have demonstrated controlled event sparsity for mobile and embedded imaging [23], and hybrid event–frame modeling and simulation have become important for realistic noise, calibration, and training of fusion algorithms [24]. The motivation is thus not only to combine two modalities but to select the right integration level per application: high-speed video reconstruction and frame interpolation require accurate temporal alignment, deblurring requires events that reliably encode motion during exposure [18,19,20], SLAM and visual odometry require geometric consistency, low latency, and robustness under high-speed motion and HDR [10,11,12], gesture recognition and always-on AIoT sensing require low power, sparse data, and event-triggered processing [13,14,25], and automotive perception requires robustness under changing illumination, HDR, long-range detection, and integration with existing stacks [21,22]. There is thus no single optimal architecture; hybrid event–frame sensing architectures should be designed through application-specific trade-offs among latency, spatial resolution, color fidelity, power, and form factor.

Previous surveys have provided broad and valuable overviews of event-based vision, including sensor principles, event representations, optical flow, image reconstruction, SLAM, and learning-based perception [8]. Other works have focused on event-camera simulators, event-based datasets, neuromorphic processors, and specific application domains such as robotics or autonomous driving [12,14,22,26,27,28,29]. However, comparatively less attention has been paid to hybrid event–frame sensing from a hardware- and system-oriented perspective for both human and machine vision. In particular, there remains a need for a review that classifies hybrid event–frame sensing architectures from a sensor-design perspective, relates those architectures to application-specific requirements, and provides practical guidelines for selecting suitable hybrid configurations. This review aims to fill that gap. The scope of this review is threefold. First, we review the operating principles and design options of hybrid event–frame sensing architectures and systems that combine event-based DVS or EVS pixels with frame-based RGB or APS pixels. The discussion covers dual-camera systems, optically aligned event–frame systems, pixel-level shared hybrid sensors, stacked or co-integrated single-chip sensors, homogeneous-pixel sensing systems, and event-only reconstruction systems. Second, we survey representative use cases in human-perceptual imaging and machine vision. Human-perceptual imaging includes high-speed imaging, video deblurring, frame interpolation, HDR imaging, low-light imaging, and AR/VR-related imaging. Machine vision includes object and person detection, gesture recognition, SLAM, visual odometry, robotics, automotive perception, and always-on AIoT sensing. Third, we analyze how application requirements should guide sensor-configuration selection. Rather than treating hybrid sensing as a single technology category, this review emphasizes that the optimal design depends on the target task, output requirement, computational budget, and deployment environment.

The remainder of this paper is organized as follows. Section 2 introduces the operating principles of frame-based RGB sensors and event-based sensors and presents a taxonomy of hybrid event–frame sensing architectures and systems, including dual-camera, optically aligned, pixel-level shared, stacked/co-integrated, homogeneous-pixel hybrid sensing, and event-only reconstruction approaches. Section 3 analyzes key sensor specifications, including latency, spatial resolution, color fidelity, power consumption, and form factor. Section 4 discusses sensor-configuration guidelines and open challenges for hybrid event–frame sensing. Section 5 provides a broader discussion of application-specific hybrid vision systems and future research directions. Section 6 concludes the paper.

To ensure consistent terminology, this review distinguishes physical event sensors, physically integrated hybrid image sensors, and broader hybrid vision systems. DVS and EVS are used synonymously in this review to denote a physical asynchronous temporal-contrast sensor or pixel that generates ON and OFF events when a local log-intensity change exceeds a predefined threshold. The combined notation DVS/EVS is used when referring generally to this class of physical event sensors without implying a difference in sensing principle. HybridEVS is reserved for RGB or Quad Bayer arrays containing embedded event pixels, particularly in the context of the MIPI HybridEVS camera pattern and its dedicated demosaicing and image-signal-processing pipeline. Pseudo-DVS denotes discrete-time event-like frames generated from a homogeneous CMOS image sensor array through high-speed sampling and temporal differencing; it does not denote a physical asynchronous address-event stream. A physically integrated hybrid image sensor incorporates frame and event sensing functions within a shared focal plane, pixel array, chip, or vertically integrated wafer stack. A hybrid vision system refers more broadly to module-level or computational combinations of frame-like and event-like information, including dual-camera systems, optically aligned systems, homogeneous-pixel sensing systems, and event-only reconstruction systems. The umbrella term hybrid event–frame sensing encompasses these module-level, physically integrated, and computationally hybrid configurations. Based on these definitions, the architectures reviewed in this paper are organized into three levels: module-level hybrid vision systems, physically integrated hybrid image sensors, and computationally hybrid vision systems.

## 2. Design and Taxonomy of Hybrid Event–Frame Sensing Architectures

In this review, a distinction is made between hybrid image-sensor hardware and the broader category of hybrid vision systems. Hybrid image-sensor hardware physically incorporates frame and event sensing functions within a shared focal plane, chip, or vertically integrated stack. By contrast, broader hybrid vision systems may combine separate camera modules, derive event-like information from a conventional frame sensor, or reconstruct frame-like images from an event-only DVS/EVS device. Accordingly, the taxonomy is organized into three levels: module-level hybrid vision systems, physically integrated hybrid image sensors, and computationally or functionally hybrid vision systems. The last category is included to cover architectures that provide complementary frame-like and event-like outputs without physically integrating distinct frame and event pixels.

### 2.1. Operating Principle of Frame-Based RGB Image Sensors

Frame-based RGB image sensors are the most widely used visual sensing devices for digital cameras, mobile imaging, surveillance, robotics, automotive cameras, and machine vision. Unlike event-based sensors, which asynchronously report brightness changes, they synchronously sample the irradiance of the entire image plane during a fixed exposure interval and output a two-dimensional frame at a fixed rate. This is most commonly implemented with CMOS active-pixel sensors (APSs), which integrate a photodiode, charge-to-voltage conversion node, pixel transistors, column readout circuits, analog-to-digital converters (ADCs), timing generators, and image signal processing (ISP) blocks on a single chip or stacked structure [1,2,3,30]. The operating principle comprises optical filtering, photoelectric conversion, charge integration, charge-to-voltage conversion, row-wise readout, digitization, and image reconstruction. Incident light first passes through a lens, an infrared-cut filter, microlenses, and a color filter array (CFA), so each pixel measures only one spectral component. The most widely used CFA is the Bayer pattern (two green, one red, one blue per 2 × 2 cell), and demosaicing is required to reconstruct a full-color image from the raw data [31,32,33]. During exposure, photons in the pinned photodiode (PPD) generate electrons that accumulate roughly in proportion to irradiance and exposure time until saturation. The charge is then transferred to a floating diffusion (FD) node, converted into a voltage, buffered by an in-pixel source follower, read through a row-select transistor, and digitized by column-parallel ADCs. Finally, an ISP pipeline performs black-level and lens-shading correction, demosaicing, white balance, color correction, gamma correction, tone mapping, denoising, sharpening, and compression [3,31,34].

**Figure 2:**
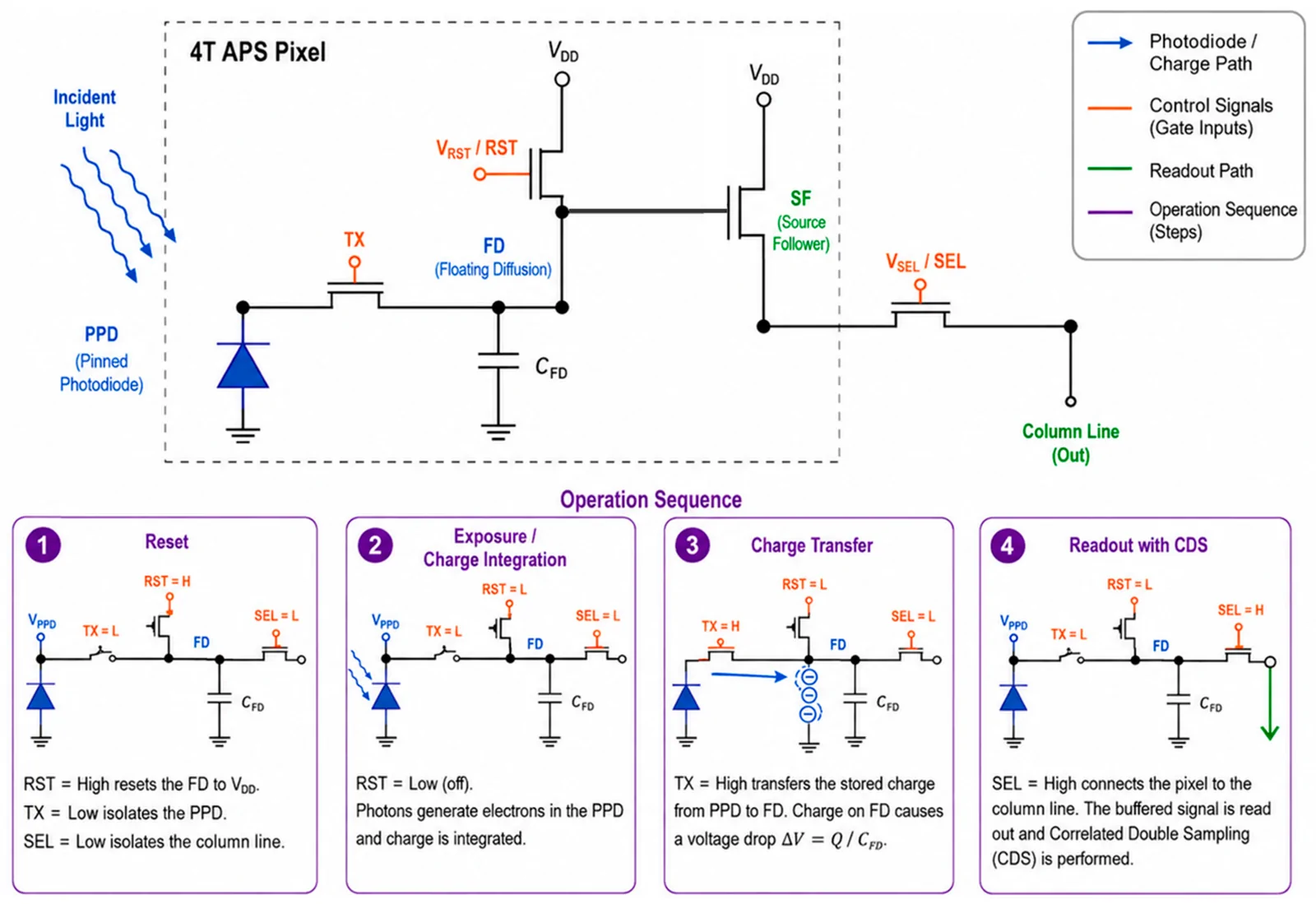
Schematic and operation sequence of a 4T active-pixel sensor (APS) with a pinned photodiode. The pixel consists of a pinned photodiode (PPD), transfer gate (TX), floating diffusion node (FD), reset transistor (RST), source follower (SF), and row-select transistor (SEL). During exposure, photo-generated charge is accumulated in the PPD. After exposure, TX transfers the charge to the FD node, where it is converted into a voltage and read out through the source follower and row-select transistor. Correlated double sampling can be performed by sampling the reset level and signal level to suppress reset noise and fixed-pattern noise. Original schematic created by the authors.

Figure 2 illustrates the basic structure and timing sequence of a four-transistor (4T) APS pixel with a pinned photodiode. The 4T APS pixel consists of a pinned photodiode (PPD), transfer gate (TX), reset transistor (RST), source follower (SF), row-select transistor (SEL), and floating diffusion capacitor (CFD). The PPD accumulates photo-generated charge during exposure. The transfer gate controls charge transfer from the PPD to the FD node. The reset transistor initializes the FD node to a reference voltage before signal readout. The source follower buffers the FD voltage, and the row-select transistor connects the pixel output to the column line when the corresponding row is selected. This 4T structure has become the dominant pixel architecture in CMOS image sensors because it enables low-noise charge transfer, high quantum efficiency, and correlated double sampling (CDS), which suppresses reset noise and fixed-pattern noise [1,2,30].

A typical 4T APS operation consists of reset, exposure, transfer, and readout phases. In reset, RST initializes the FD node and the reset voltage is sampled as a reference. During exposure, RST and TX are off and photons are integrated in the PPD. At the end of exposure, TX transfers the accumulated charge to the FD node, and the FD voltage change represents the photo-signal. During readout, SEL turns on and the source follower drives the column line; the column circuit samples both reset and signal levels, and CDS subtracts them to reduce kTC reset noise and pixel offset. This low-noise operation is a key reason PPD-based 4T APS pixels are widely used in modern CMOS image sensors [1,2].

The pixel output voltage (*V_sig_*) can be expressed in simplified form as

**Figure 3:**
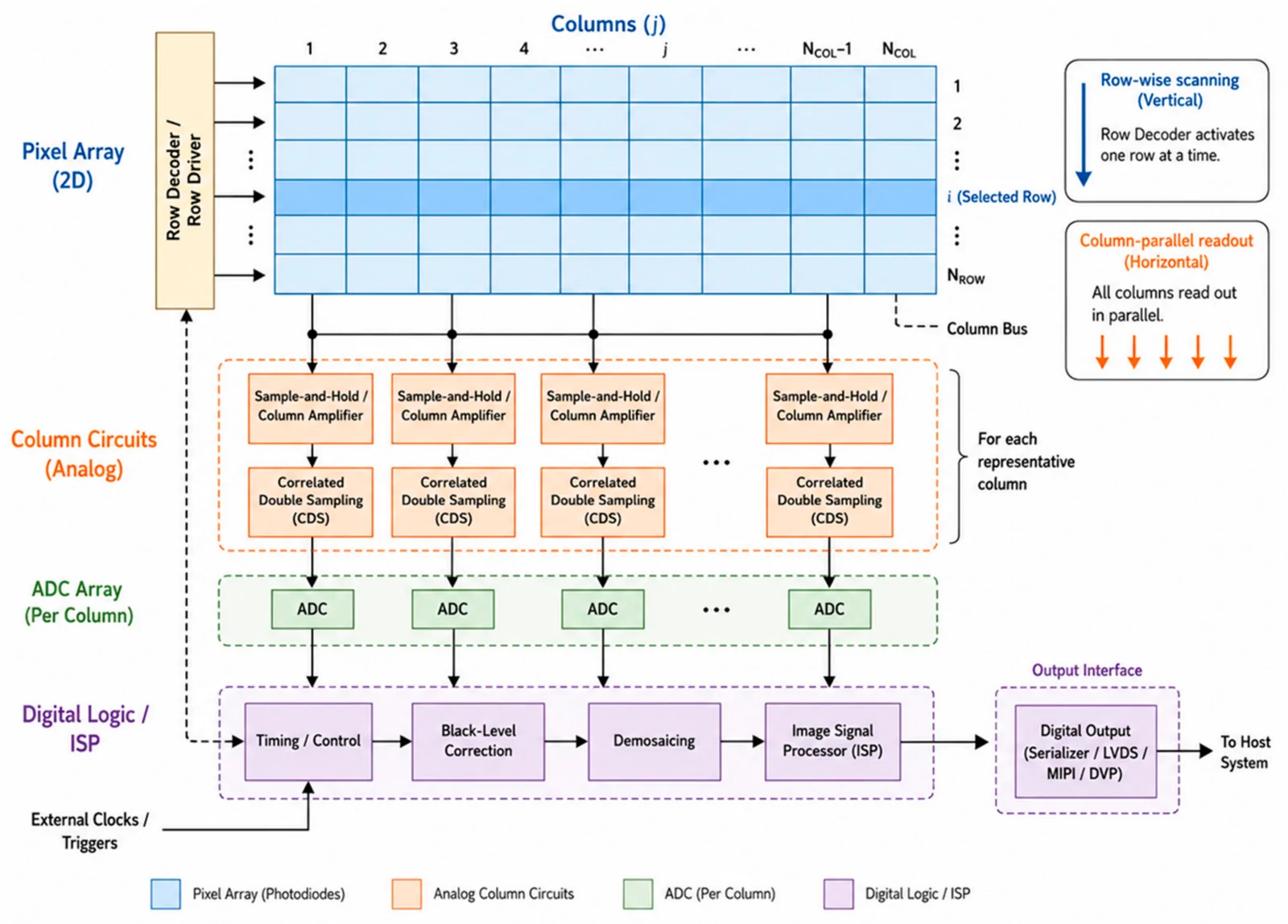
Typical column-parallel readout architecture of a frame-based CMOS image sensor. The pixel array is read row by row. Each selected pixel drives its column line through a source follower, and each column includes a sampling circuit, correlated double sampling (CDS), amplifier, and ADC. The digitized raw data are processed by digital blocks and ISP circuits. Column-parallel readout enables high-resolution and high-frame-rate imaging by performing analog-to-digital conversion in parallel across columns. Original schematic created by the authors.

*V_sig_* = *V_rst_* − *Q_ph_*/*C_FD_* (1)

where *V_rst_* is the reset voltage of the FD node, *Q_ph_* is the photo-generated charge transferred from the photodiode, and *C_FD_* is the floating diffusion capacitance. The conversion gain is inversely proportional to *C_FD_*. A smaller FD capacitance increases conversion gain and improves low-light sensitivity, but it can reduce full-well capacity and dynamic range. Conversely, a larger FD capacitance increases charge capacity but reduces voltage swing per electron. Therefore, APS pixel design requires careful trade-offs among sensitivity, full-well capacity, dynamic range, noise, pixel pitch, and readout speed [1,2,3,30]. Figure 3 shows a simplified column-parallel readout architecture for a frame-based CMOS image sensor. Pixels are arranged in a two-dimensional array and are usually read row by row. When a row is selected, each pixel in that row drives its corresponding column line. Each column includes analog sampling circuits, CDS circuits, amplifiers, and ADCs. Column-parallel readout is essential for high-resolution and high-frame-rate image sensors because it distributes the analog-to-digital conversion across many columns instead of using a single global ADC. This reduces readout bottlenecks and enables high-throughput image acquisition with manageable power consumption [3,30,35].

Modern RGB image sensors often adopt stacked CMOS architectures to improve performance and integration density. The pixel array is fabricated on an imaging wafer, while logic, ADCs, memory, and image-processing circuits are fabricated on a separate logic wafer and connected through wafer bonding or through-silicon vias, letting the pixel layer be optimized for optical performance and the logic layer for high-speed processing, memory bandwidth, and low power. Recent high-resolution stacked sensors achieve high pixel counts, small pixel pitch, multiple sampling, and high-speed readout by combining advanced pixels with fine-pitch stacking and column-parallel processing [35]. Frame-based RGB sensors offer dense spatial information for texture, shape, color, and semantic interpretation; compatibility with mature ISP pipelines and computer-vision algorithms; visually interpretable images for human viewing or deep-network input; and high spatial resolution and color fidelity. However, they also have limitations that motivate hybrid sensors. Fixed-rate sampling generates redundant data for static scenes and may miss fast motion at insufficient frame rates. Motion during exposure causes blur, while short exposures used to suppress blur degrade the signal-to-noise ratio in low light. Higher frame rate or resolution raises bandwidth, memory, and power, and a single exposure may saturate bright regions or lose dark detail in high-dynamic-range scenes. These limitations are critical in high-speed imaging, AR/VR, robotics, automotive perception, and always-on edge-AI, where latency, dynamic range, and energy efficiency matter.

### 2.2. Operating Principle of Event-Based Sensors

Event-based image sensors, generally referred to as dynamic vision sensors (DVSs), event vision sensors (EVSs), or event cameras, operate according to a fundamentally different sensing principle from conventional frame-based RGB image sensors. While a frame-based sensor integrates light over a fixed exposure time and outputs a synchronous two-dimensional image, an event-based sensor asynchronously detects temporal brightness changes at each pixel and outputs sparse address-events only when a local change occurs [5,6,7,8,9]. This sensing principle enables microsecond-level temporal resolution, low latency, high dynamic range, and reduced redundant data output, making event-based sensors suitable for high-speed motion analysis, robotics, SLAM, gesture recognition, automotive perception, and always-on edge-AI sensing [8,11,13,25,27].

The basic output of an event-based sensor is an event stream rather than a frame sequence. Each event can be represented as

*e_k_* = (*x_k_*, *y_k_*, *t_k_*, *p_k_*) (2)

where *x_k_* and *y_k_* denote the pixel address, *t_k_* is the timestamp, and *p_k_* is the event polarity. A positive event, or ON event, is generated when the logarithmic brightness at a pixel increases by more than a positive contrast threshold. A negative event, or OFF event, is generated when the logarithmic brightness decreases by more than a negative contrast threshold. If *L*(*x*,*y*,*t*) = *log I*(*x*,*y*,*t*) denotes the logarithmic irradiance at a pixel, an event is generated when

*L*(*x*, *y*, *t_k_*) − *L*(*x*, *y*, *t_k_* − Δ*t*) ≥ *C_ON_* (3)

or

**Figure 4:**
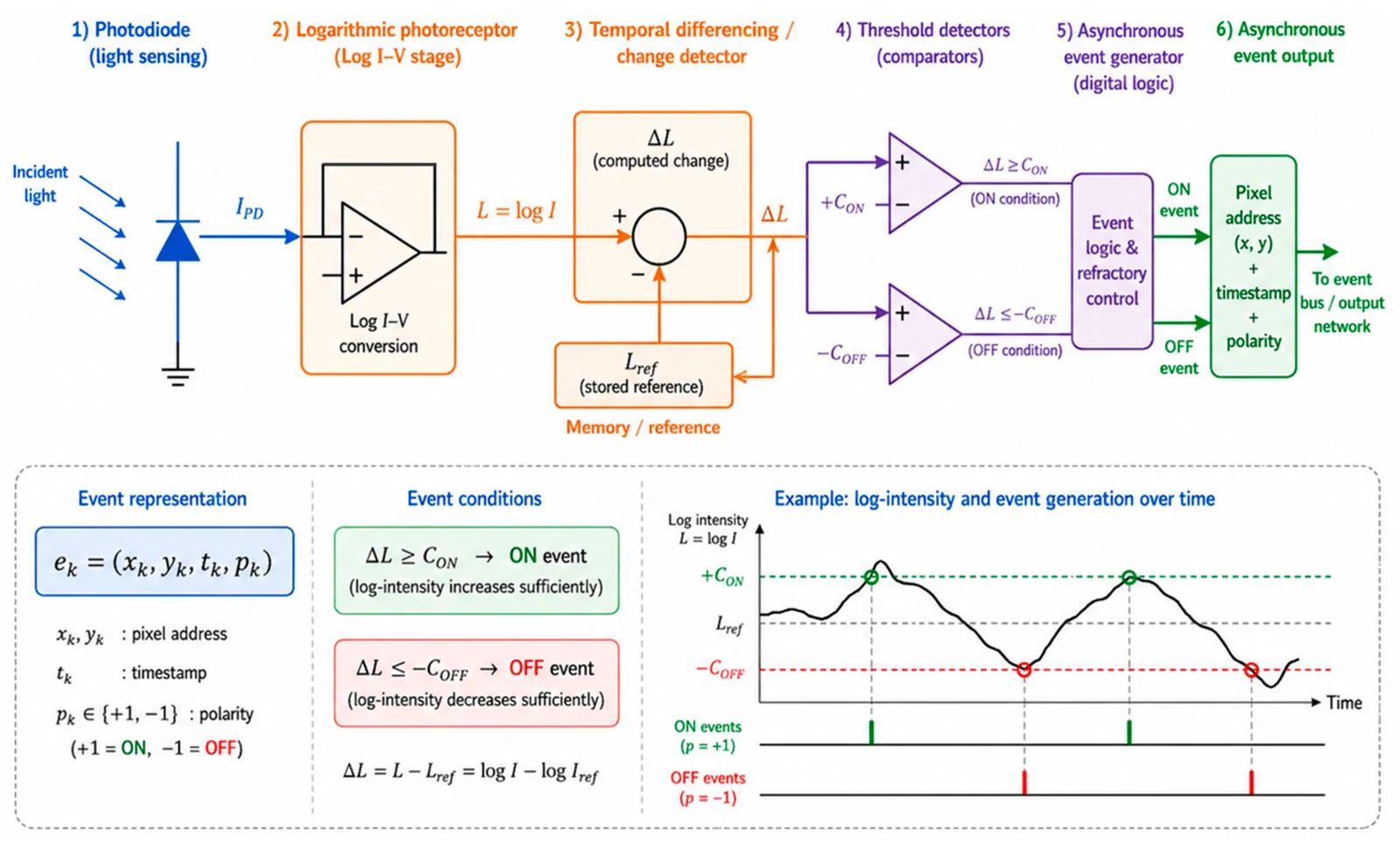
Simplified DVS pixel structure and event-generation principle. A DVS pixel converts incident light into photocurrent using a photodiode. The logarithmic photoreceptor transforms the photocurrent into a log-intensity voltage. The temporal differencing circuit compares the current log-intensity signal with a stored reference level. When the difference exceeds the positive or negative contrast threshold, the comparator generates an ON or OFF event. The output event contains the pixel address, timestamp, and polarity. Original schematic created by the authors.

*L*(*x*, *y*, *t_k_*) − *L*(*x*, *y*, *t_k_* − Δ*t*) ≤ −*C_OFF_* (4)

where *C_ON_* and *C_OFF_* are the ON and OFF contrast thresholds, respectively. Therefore, a DVS does not measure absolute intensity directly, but reports local temporal contrast. This log-intensity change detection makes the sensor naturally robust to large illumination variations and enables very high dynamic range compared with conventional fixed-exposure frame sensors [5,6,36,37,38]. A typical event pixel consists of a photodiode, a logarithmic photoreceptor, a temporal differencing circuit, a pair of comparators or thresholding circuits, and an event-generation interface as shown in Figure 4. The photodiode converts incident photons into photocurrent. The logarithmic photoreceptor converts the photocurrent into a voltage proportional to the logarithm of the input intensity. This logarithmic response is important because it allows the pixel to detect relative brightness changes rather than absolute intensity changes. The temporal differencing circuit stores or tracks a previous reference level and compares it with the current log-intensity signal. When the difference exceeds the ON or OFF threshold, the comparator generates a polarity event. After event generation, the reference level is updated so that the pixel can detect the next brightness change [5,6,7].

**Figure 5:**
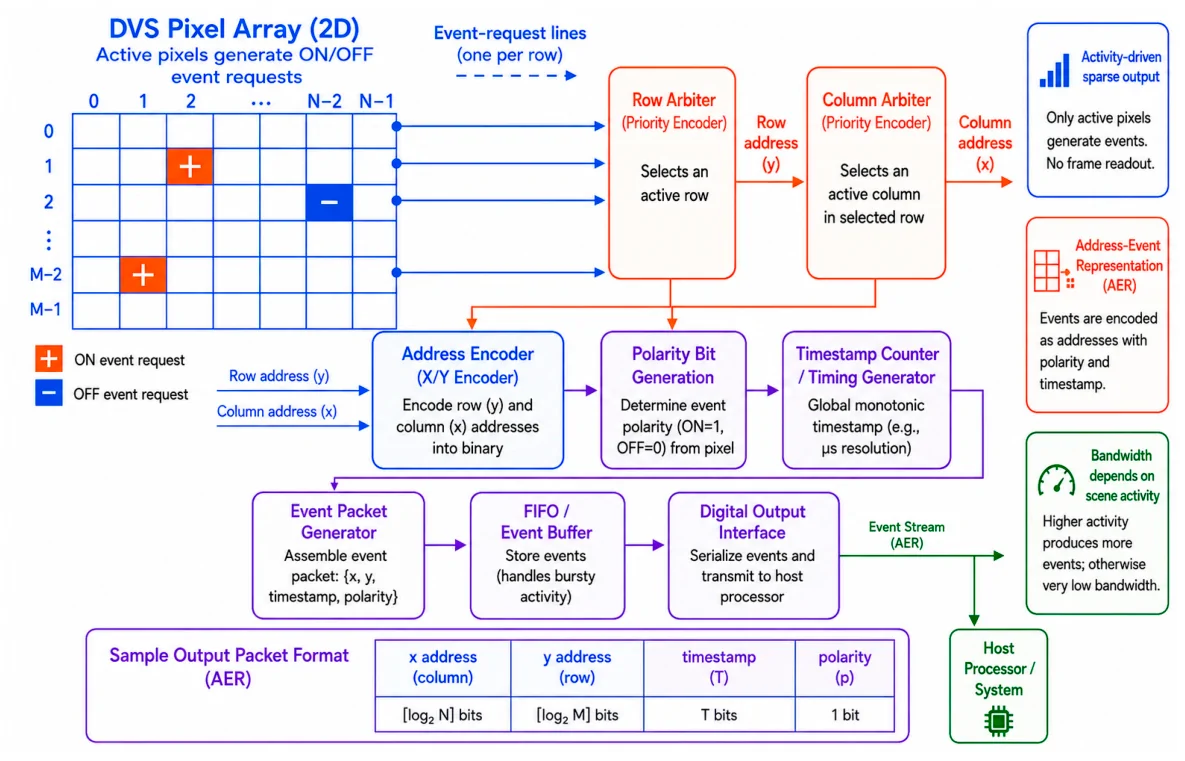
Typical readout architecture of an event-based DVS. Pixels asynchronously generate ON or OFF event requests when the local log-intensity change exceeds a contrast threshold. Row and column arbiters select active pixels, encode their addresses, and combine the address with polarity and timestamp information. The resulting event packets are transmitted as an asynchronous event stream. Unlike frame-based readout, the bandwidth of this architecture depends mainly on scene activity and event rate. Original schematic created by the authors.

The operation of a DVS pixel can be divided into four steps: photoreception, logarithmic conversion, temporal contrast detection, and asynchronous event transmission. Incident photons generate photocurrent in the photodiode; the photoreceptor converts it into a logarithmic voltage; the pixel detects the change relative to a stored reference; and, if the change exceeds the threshold, an ON or OFF event is produced and transmitted through an address-event representation (AER) interface [5,7,39]. The AER scheme is a key difference from frame-based sensors. Whereas a CMOS image sensor reads pixels row by row at a fixed rate regardless of activity, a DVS reads out only pixels that generate events: a pixel detecting a change requests row and column arbitration, and the peripheral logic encodes its address, polarity, and timestamp into an event packet. Since only active pixels are read out, output bandwidth scales with scene dynamics rather than resolution [5,6,7], which is useful for low-power edge sensing because static backgrounds do not continuously generate data. Figure 5 shows a typical DVS readout architecture, in which row and column arbiters resolve simultaneous ON/OFF requests, encode the active-pixel address, append a polarity bit and timestamp, and transmit the event stream to the host or edge-AI accelerator. In high-resolution designs, arbitration speed, event congestion, timestamp precision, and readout-induced motion artifacts become important. Suh et al. reported a 1280 × 960 DVS with a 4.95 μm pixel pitch and a readout scheme that minimizes motion artifacts, showing that readout architecture is critical for practical high-resolution event sensing [40].

The advantages of event-based sensing arise directly from this operating principle. Event cameras achieve very low latency because events are generated immediately after local brightness changes, without waiting for the next frame; they capture high-speed motion because timestamp resolution is much finer than the frame interval; they provide high dynamic range because the logarithmic photoreceptor responds to relative changes over a wide illumination range; and they reduce redundant data because static regions produce few events. These properties make them attractive for high-speed imaging, motion deblurring, visual odometry, SLAM, gesture recognition, robotics, and automotive perception [8,11,13,18,19,20,25,27]. However, event-based sensors also have limitations. Because DVS pixels detect changes rather than absolute intensity, they do not directly provide dense grayscale or RGB images, and static or textureless regions generate few events, making purely event-based recognition difficult. Event generation depends on contrast threshold, bias currents, illumination, device mismatch, and noise, and background-activity events may appear even in static scenes due to leakage, thermal noise, dark current, and mismatch [15,16,17]. Event streams are also sparse, asynchronous, and irregularly sampled, requiring specialized representations and algorithms [8,41]. These characteristics make event and frame sensing complementary: in hybrid sensing, the event output supplies motion cues, temporal alignment, and blur compensation, while the frame output preserves color fidelity and human-interpretable quality. From a design viewpoint, DVS pixels require extra circuits (logarithmic photoreceptors, temporal contrast detectors, comparators, request circuits, and asynchronous readout), increasing pixel complexity and reducing fill factor or resolution, and high-resolution designs must manage event bandwidth, timestamp precision, arbitration latency, and motion artifacts. Conversely, sparse asynchronous output greatly reduces bandwidth and power when activity is low. Event-based sensors therefore require a different optimization strategy, jointly balancing contrast sensitivity, latency, event rate, noise, timestamp accuracy, readout bandwidth, and power.

Table 1 summarizes the fundamental differences among frame-based RGB sensors, event-based sensors, and hybrid event–frame systems. Frame-based RGB sensors provide dense, color-rich, human-interpretable images, but synchronous exposure and readout impose trade-offs among frame rate, motion blur, bandwidth, power, and dynamic range. Event-based sensors address several of these by generating sparse asynchronous events only when local logarithmic brightness changes exceed a threshold, enabling low-latency, high-dynamic-range sensing, but pure event streams lack dense color, texture, and static scene information. Hybrid sensors combine the two: the RGB or APS path provides spatially dense, color-rich information while the DVS or EVS path provides fast temporal change, making hybrid sensing attractive when both human-perceptual image quality and machine-vision robustness are required. However, the best architecture depends on the application, because dual-camera, optically aligned, pixel-level shared, stacked, homogeneous-pixel hybrid sensing, and event-only reconstruction involve different trade-offs in synchronization, calibration, pixel area, readout bandwidth, power, and form factor.

### 2.3. Dual-Camera Systems

**Figure 6:**
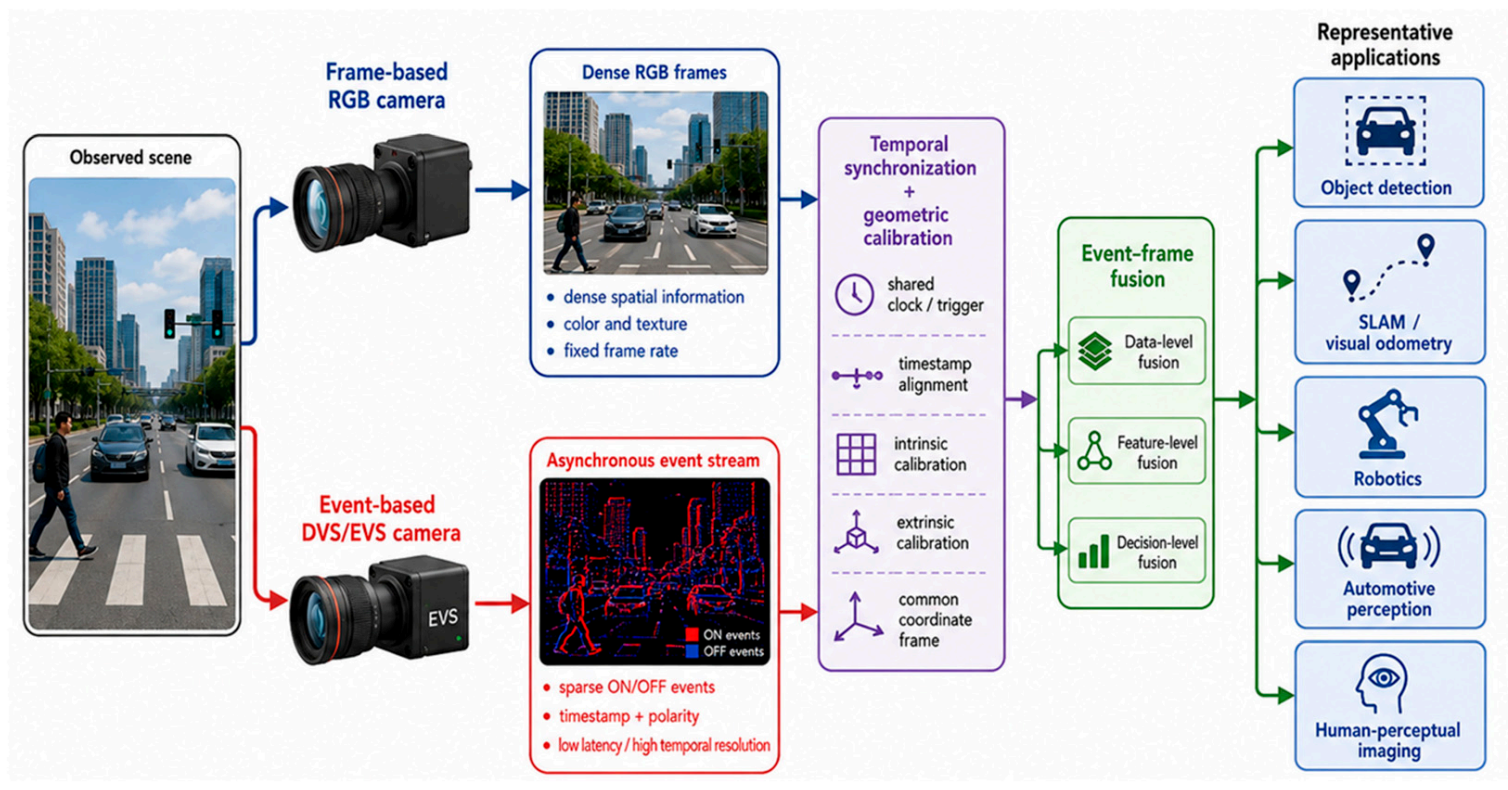
Conceptual architecture of a dual-camera event–frame system. A frame-based RGB camera and an event-based DVS/EVS camera are mounted as separate modules and observe the same scene through independent optical paths. The RGB camera provides dense color frames, while the event camera provides sparse asynchronous events. Temporal synchronization and geometric calibration are required before event–frame fusion. The two modalities can be fused at the data level, feature level, or decision level depending on the target application. Original schematic created by the authors.

A dual-camera event–frame system is the simplest and most flexible architecture for combining frame-based RGB imaging and event-based sensing. In this configuration, an RGB camera and an event camera are mounted as two physically separate imaging modules. Each camera has its own lens, sensor, readout circuit, timing interface, and data path. The RGB camera captures dense color or grayscale frames at a fixed frame rate, while the event camera independently produces asynchronous ON/OFF events according to local brightness changes. Compared with pixel-level or chip-level hybrid sensors, the dual-camera approach does not require special semiconductor integration. For this reason, dual-camera systems have been widely used in early-stage event–frame research, dataset construction, algorithm prototyping, object detection, visual odometry, stereo vision, and autonomous-driving experiments [8,12,21,22,42,43]. Therefore, it is particularly useful when the objective is to evaluate event–frame fusion algorithms rather than to develop a new image sensor chip. Figure 6 illustrates the conceptual architecture of a dual-camera event–frame system. Once calibrated, RGB frames and event streams can be projected into a common coordinate frame and fused at the data, feature, or decision level.

The main advantage of the dual-camera architecture is modularity. Since each camera is independent, the system can be configured for various applications without redesigning the sensor hardware. DSEC is a representative example: it provides stereo data from two high-resolution monochrome event cameras and two global-shutter color cameras, together with LiDAR and RTK GPS measurements, for driving scenarios under different lighting conditions [22]. Tomy et al. proposed an event–frame fusion approach for object detection in which features from the RGB camera and event camera are combined to improve robustness against image corruptions and difficult weather or lighting conditions [44]. In this type of system, RGB frames provide rich semantic and texture information, whereas events provide edge and motion cues with high temporal resolution that remain useful when frame images are degraded by blur, overexposure, underexposure, or rapid motion. Therefore, dual-camera fusion can improve machine-vision robustness without requiring a fully integrated hybrid sensor. Event–frame fusion can therefore improve state estimation robustness by exploiting the complementary operating regimes of the two modalities [11,45,46].

Despite its flexibility, the dual-camera approach has several limitations. First, because the two cameras use separate lenses and are located at different physical positions, their images are affected by parallax. Even after calibration, a point in the scene may project to different positions depending on depth. This makes pixel-level alignment difficult, especially for nearby objects, wide-baseline setups, or scenes with strong depth variation. Second, the two cameras may have different fields of view, resolutions, optical distortions, exposure times, timestamps, and rolling- or global-shutter characteristics. These differences complicate event–frame fusion. Third, mechanical vibration, temperature drift, and lens changes can degrade calibration accuracy over time. Fourth, the dual-camera module is larger and more power-consuming than a single-chip hybrid sensor. These limitations are especially important for mobile devices, AR/VR headsets, drones, wearable cameras, and always-on AIoT systems, where compact form factor and low power are critical. The level at which fusion is performed is also important. In data-level fusion, events are accumulated over a temporal window and projected onto the RGB image plane to form event images, time surfaces, or motion maps. This approach is simple and compatible with convolutional neural networks, but it may lose the asynchronous temporal precision of individual events. In feature-level fusion, separate encoders extract features from RGB frames and event representations, and the features are fused through concatenation, attention, recurrent processing, or transformer-based architectures. This approach can better preserve modality-specific information but requires more training data and computational resources. In decision-level fusion, RGB-based and event-based predictions are produced independently and then combined. This is robust and modular, but it may not fully exploit low-level correlations between events and frames [40,44,47,48].

From a hardware perspective, dual-camera systems are most suitable for research platforms, dataset collection, autonomous-driving test vehicles, robotics platforms, and industrial systems in which module size and calibration complexity are acceptable. They are less suitable for highly compact consumer devices, mobile cameras, and ultra-low-power always-on systems. Nevertheless, dual-camera systems remain important because they provide a practical and flexible platform for exploring event–frame complementarity before sensor-level integration.

### 2.4. Optically Aligned Event–Frame Systems

**Figure 7:**
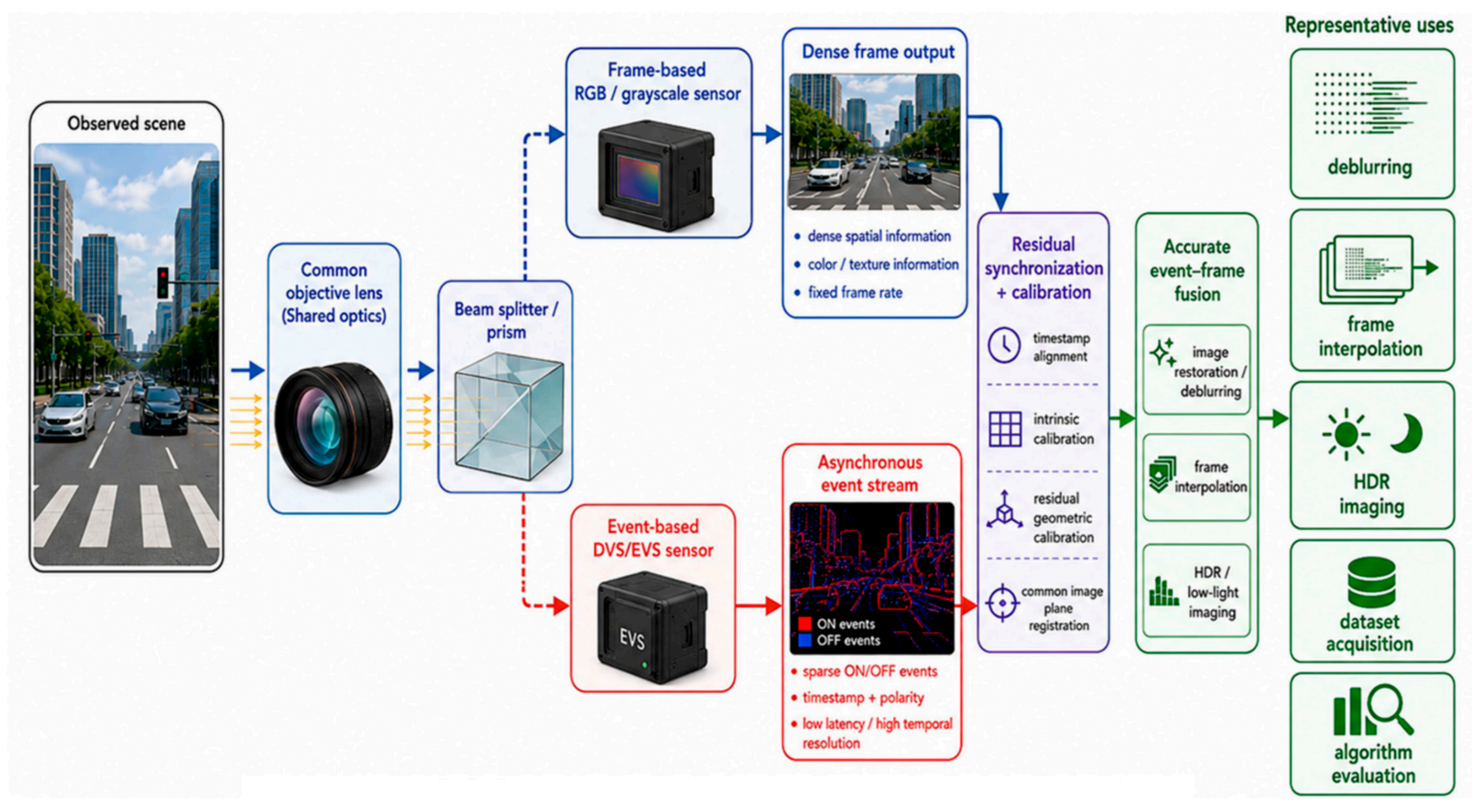
Conceptual architecture of an optically aligned event–frame system. A common objective lens collects light from the scene, and a beam splitter or prism divides the optical path into a frame-based RGB or grayscale sensor and an event-based DVS/EVS device. The RGB or grayscale sensor provides dense image frames, while the event sensor provides sparse asynchronous ON/OFF events. Since the two sensors share nearly the same optical axis, parallax is reduced and event–frame registration becomes more accurate than in a conventional dual-camera system. Original schematic created by the authors.

Optically aligned event–frame systems represent a more tightly coupled architecture than loosely mounted dual-camera systems. In a conventional dual-camera configuration, the RGB and event cameras observe the scene from slightly different viewpoints because of separate lenses and physically separated optical centers, causing parallax and difficult pixel-level alignment for nearby objects, wide baselines, and large depth variation. Optically aligned systems address this with a shared optical path, beam splitter, prism, or co-axial arrangement so that both sensors receive light from nearly the same viewpoint. As a result, events and frames can be more accurately registered in the image plane, which is particularly important for image restoration, video reconstruction, frame interpolation, deblurring, HDR imaging, and dense fusion [8,19,20,49,50,51]. Figure 7 illustrates this structure: incident light is collected by a common objective lens, then divided by a beam splitter or prism into two paths, one to the frame-based RGB or grayscale sensor and one to the event-based DVS/EVS device. The frame sensor outputs dense frames at a fixed rate while the event sensor outputs asynchronous ON/OFF events with high temporal resolution. Because both share nearly the same optical axis, the event stream can be projected onto the frame image plane with reduced parallax; temporal synchronization and residual geometric calibration are still required, but the calibration problem is significantly simplified because the modalities are optically co-located.

The most important advantage of optical alignment is improved spatial correspondence between events and frames. In event-guided image restoration, each event should correspond to the same scene point observed by the frame sensor. If the event camera and frame camera are separated by a baseline, depth-dependent parallax may cause the event edges to be misaligned with image edges. In contrast, a beam-splitter configuration can reduce this geometric inconsistency by making the two modalities nearly coaxial. This is why optically aligned event–frame systems have been widely used in laboratory proto-types, dataset acquisition, and algorithm evaluation for event-guided imaging tasks [19,20,50,51,52,53]. A representative application is event-guided video deblurring. During a long exposure, a frame-based image sensor integrates light over time, and motion during the exposure produces a blurry image. An event camera observing the same scene provides brightness-change information with high temporal resolution during the exposure interval. The blurry frame can then be modeled as the temporal integration of latent sharp images, while the event stream provides constraints on how the latent image changes over time. Pan et al. proposed the Event-based Double Integral (EDI) model, which uses a blurry frame and corresponding events to reconstruct high-frame-rate sharp video [19,50]. Time Lens and related methods use events between frames to synthesize intermediate images with improved temporal accuracy [20]. Recent event-guided low-light and image-restoration studies have therefore used event–frame fusion to improve visibility, motion sharpness, and temporal consistency [52,53,54].

Despite these advantages, optically aligned event–frame systems have several optical and calibration limitations. Beam splitters reduce the photon flux delivered to each sensor; for example, an ideal 50:50 splitter provides approximately half of the incident optical power to each path, which can degrade the signal-to-noise ratio under low-light conditions. Practical beam splitters and prisms also exhibit non-ideal transmission and reflection characteristics that depend on wavelength, polarization, incidence angle, coating design, and optical geometry. Differences between the s- and p-polarized transmission or reflection responses can produce path-dependent radiometric variations, while wavelength-dependent splitting can cause spectral imbalance between the RGB frame and event paths. The latter may alter RGB color reproduction and the effective brightness contrast detected by the event sensor. In addition, multiple reflections at coated or uncoated optical interfaces can generate flare and ghost images. Such ghosts may appear as displaced or attenuated structures in the frame image and may generate spurious or spatially shifted events in the event path, thereby degrading event–frame correspondence and fusion accuracy. Therefore, geometric and temporal calibration alone may not be sufficient; practical systems may also require path-specific radiometric and spectral calibration, characterization of polarization sensitivity, and optical measures such as antireflection coatings, baffling, or ghost-suppression design. Registration is improved but not eliminated because the two sensors may still differ in resolution, spectral response, pixel pitch, distortion, exposure timing, and readout latency. The approach also increases optical complexity and module volume. Thus, optically aligned systems are best suited to research platforms, high-quality dataset acquisition, controlled imaging, and applications where accurate event–frame correspondence is more important than form factor.

### 2.5. Pixel-Level Shared Hybrid Sensors

Pixel-level shared hybrid sensors represent one of the earliest and most influential forms of event–frame integration. A representative example is the DAVIS family of sensors, which integrates APS and DVS functionality within the same pixel array and shares a common optical path for both modalities [6,7]. The key characteristic of a DAVIS-type hybrid sensor is that frame and event information originate from the same pixel location. This property is especially valuable for applications that require precise spatiotemporal association, such as visual odometry, SLAM, motion estimation, object tracking, and event-guided image reconstruction [7,8,9].

**Figure 8:**
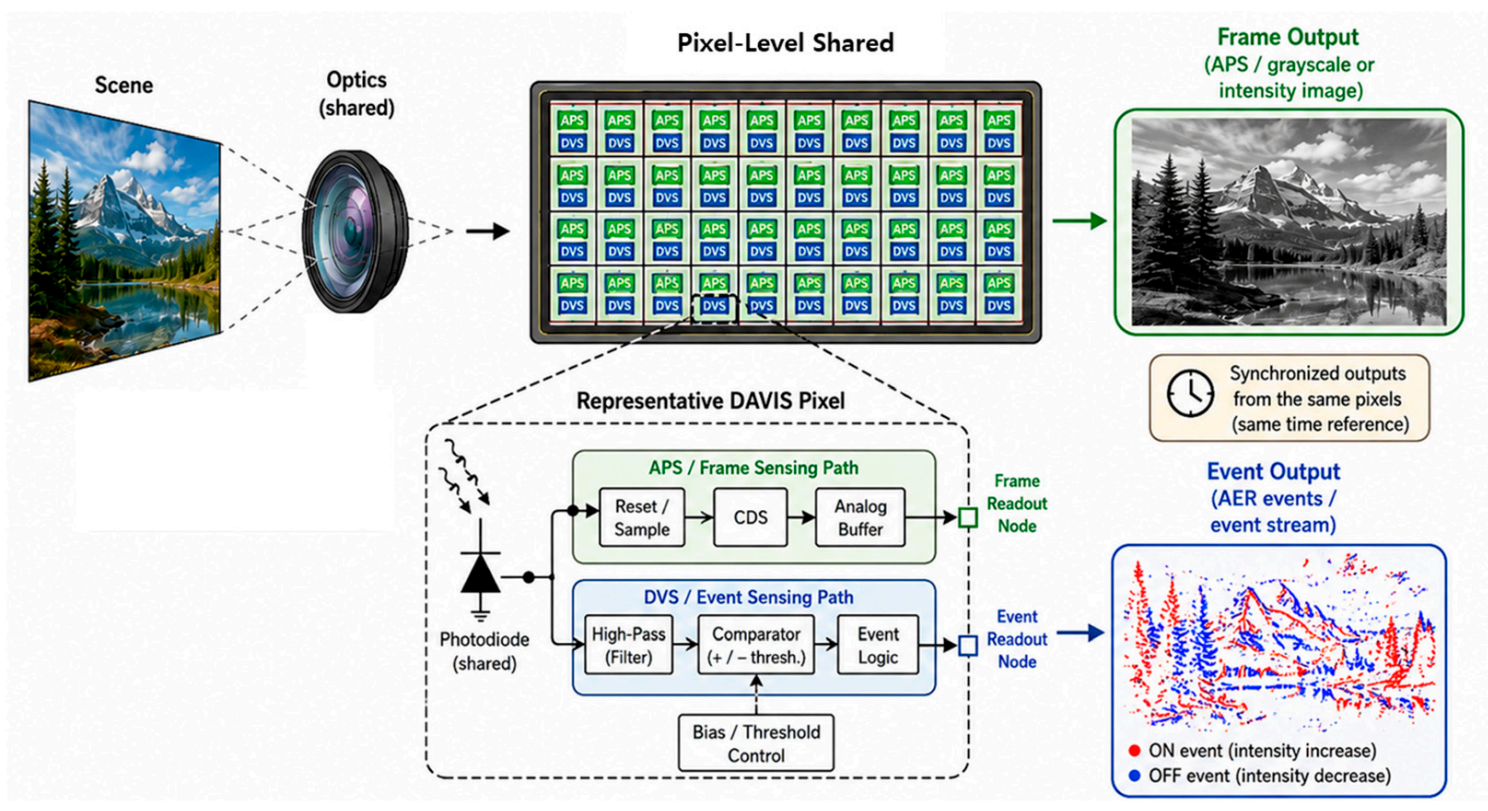
Conceptual structure of a pixel-level shared hybrid image sensor. Incident light is captured by a shared photodiode at each pixel. The photodiode output is split into two parallel sensing paths: an APS path that produces frame-based intensity images and a DVS path that generates asynchronous ON/OFF events. Because both outputs originate from the same pixel array and the same optical path, the sensor provides intrinsic spatial alignment between frame and event data. Original schematic created by the authors.

As illustrated in Figure 8, incident light is collected by a shared photodiode, and the resulting photocurrent is routed to two parallel sensing paths. One path is used for conventional APS frame readout, while the other path is used for DVS event generation. In the APS path, the photodiode signal is sampled and read out in a frame-based manner through standard pixel circuitry and readout logic. This path provides absolute intensity information at a fixed frame rate, typically in grayscale form in early DAVIS implementations, although color extensions are also possible. When the change in log intensity exceeds a positive or negative threshold, the event circuitry generates an ON or OFF event and transmits it through an address-event representation (AER) interface [7,9]. The frame path is well suited for scene interpretation, low-frequency texture capture, and human-readable imaging, whereas the event path is advantageous for low-latency motion sensing, high-speed dynamics, and reduced redundant data transmission [8,9]. In addition, the availability of both frame and event outputs from the same sensor has made DAVIS-type devices a standard platform for developing and benchmarking event-based vision algorithms [7,8].

Despite these advantages, pixel-level hybrid integration introduces important trade-offs. First, because APS and DVS circuitry must coexist within the pixel array, the pixel architecture becomes more complex than a pure frame or event sensor, and the added transistors, routing, and readout circuits can reduce fill factor and increase pixel pitch, limiting spatial resolution. Second, supporting both frame and event readout complicates the peripheral architecture and may raise power and bandwidth requirements. Third, color capability is limited in many implementations: most widely used DAVIS sensors are monochrome, and extending the concept to color introduces challenges in color-filter arrangement, sensitivity loss, and pixel-layout complexity [7,9].

From a system perspective, DAVIS-type sensors are particularly suitable when accurate event–frame alignment matters more than maximizing standalone frame resolution or minimizing pixel complexity; their strength is tightly coupled sensing rather than independent optimization of each modality. Compared with dual-camera systems they offer better spatial consistency but less flexibility, and compared with stacked hybrids, they are conceptually simpler and historically earlier but less favorable for very high resolution or advanced color imaging, because the hybrid functionality must fit within the focal-plane pixel array itself.

### 2.6. Stacked or Co-Integrated Single-Chip Sensors

**Figure 9:**
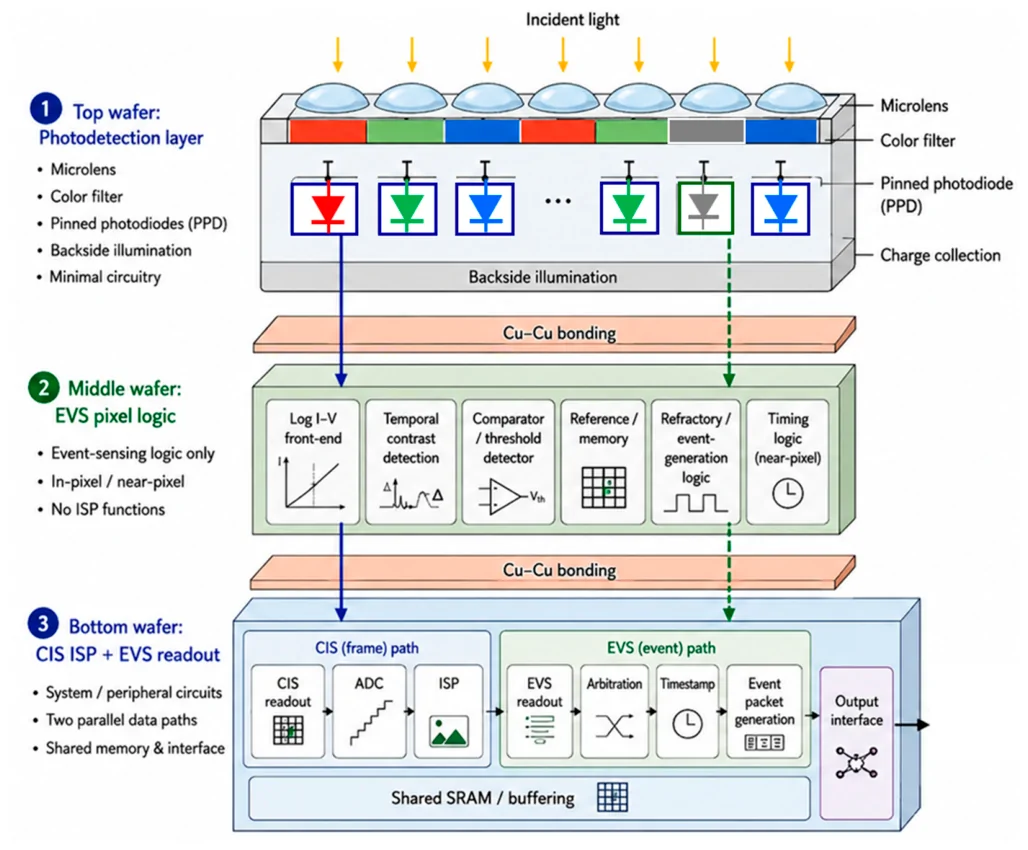
Cu–Cu-bonded three-wafer-stacked CIS–DVS hybrid image sensor architecture. The top wafer is the photodetection layer and contains microlenses, color filters, backside-illuminated pinned photodiodes, and charge-collection structures. The middle wafer implements DVS/EVS pixel logic, including temporal contrast detection and event-generation-related circuits. The bottom wafer integrates the CIS frame-processing path and the DVS/EVS event-readout path, including frame readout, ISP, event arbitration, timestamping, shared buffering, and output interface circuits. Cu–Cu direct bonding provides high-density vertical electrical interconnects among the three wafers. The microlenses and photodiodes are shown schematically; practical heterogeneous RGB–event arrays may require pixel-type- and field-position-dependent microlens profiles and CRA shifts, together with crosstalk-aware calibration. Original schematic created by the authors.

Stacked or co-integrated single-chip sensors represent a highly integrated hardware approach for hybrid image sensing. This approach is particularly attractive for hybrid CIS–DVS image sensors, because frame-based imaging and event-based sensing require different circuit functions, bandwidth, and timing characteristics [55,56,57,58]. In this review, stacked or co-integrated hybrid sensors are discussed specifically in the context of Cu–Cu-bonded three-wafer-stacked CIS–DVS architectures, as illustrated in Figure 9. In such a structure, the three wafers are functionally separated as follows: (1) a top photodetection wafer containing pinned photodiodes (PPDs), (2) a middle wafer implementing DVS pixel logic, and (3) a bottom wafer integrating CIS ISP and DVS readout circuits. It also differs from planar hybrid arrays because the key design principle is not lateral pixel placement, but vertical allocation of sensing, analog/event logic, and digital/system functions across different bonded wafers.

Figure 9 conceptually summarizes this architecture. Incident light passes through microlenses and the color filter array into the top wafer, where PPDs convert photons into charge. This top wafer is the primary photodetection layer, optimized for backside illumination, optical efficiency, color sampling, charge collection, and pixel pitch, retaining only minimal circuitry so optical aperture and quantum efficiency are preserved [55,59]. The middle wafer implements DVS pixel logic—logarithmic current-to-voltage conversion, temporal contrast detection, comparator thresholding, local reference/memory, refractory or event-generation logic, and near-pixel timing—transforming the top-layer PPD signal into event-domain information; placing it on a separate wafer avoids consuming optical area [55,56]. The bottom wafer is the digital and system layer, providing frame readout, ADCs, ISP, memory, buffering, and output interfaces for the CIS path and event readout, arbitration, timestamping, packet generation, compression, and output control for the DVS path, optionally with shared SRAM and event signal processing (ESP). It thus supports two parallel but coordinated paths—a CIS frame path for dense imaging and an event path for asynchronous change reporting—so conventional image output and low-latency event output coexist in one compact device [55,56]. A representative example is the three-wafer-stacked hybrid 15 Mp CIS + 1 Mp DVS reported by Guo et al. [55,56], integrating a 4096 × 3680 CIS and a 1032 × 928 DVS, supporting 4.6 GEvents/s readout, and incorporating in-pixel time-to-digital conversion (TDC), on-chip ISP, and ESP, making it a clear example of a vertically co-integrated hybrid sensor rather than a juxtaposition of frame and event circuits [55].

The architectural advantage of the three-wafer-stacked CIS–DVS sensor follows from the distinct needs of frame and event vision. The CIS path requires high-quality photodetection, stable frame readout, ADC operation, and ISP to preserve resolution, tone, and color, whereas the DVS path requires fast temporal differencing, local thresholding, timestamping, and high-throughput sparse readout. Implementing all of this in a single planar layer would severely constrain photodiode area, fill factor, and routing; the three-wafer-stacked structure separates these roles—top wafer for optical performance, middle for event-detection logic, bottom for peripheral and digital circuits—improving functional density without forcing all circuitry into one-pixel footprint [55,56,57,58]. Cu–Cu direct bonding is a critical enabler, providing fine-pitch, low-resistance, high-density vertical interconnections that transfer signals among wafers without excessive parasitics or area overhead, so it is not merely packaging but a key architectural enabler for vertically integrated hybrid sensing [55,57]. The stack also enables parallel yet coordinated processing: the CIS and DVS paths coexist within one stack and can share memory, timing, and output infrastructure, reducing off-chip bandwidth, improving synchronization, and simplifying integration. From a common optical front end, the sensor thus provides dense frames for human-perceptual imaging and sparse events for low-latency motion sensing [55,56].

In heterogeneous stacked CIS–DVS sensors, however, sharing a lens and a nominally common optical axis does not necessarily guarantee identical optical acquisition in the frame and event paths. Depending on the implementation, RGB and event photodetection sites may differ in pixel pitch, photodiode geometry or depth, color-filter stack, and microlens profile. Because the optimum lateral shift in the microlens and optical stack depends on the chief-ray angle (CRA) and pixel geometry, particularly for off-axis pixels, an optical design optimized for RGB sites may produce a different angular response, photon-collection efficiency, and lens-shading characteristic at event sites [59]. Pixel-level optical crosstalk can further couple incident light between neighboring RGB and event sites. In the frame path, this can cause color mixing and spatial blur, whereas in the event path it can attenuate or spatially spread the local temporal contrast, thereby shifting threshold-crossing time and altering event density or edge localization [60]. Therefore, heterogeneous stacked sensors require pixel-type-aware CRA and microlens co-design, optical isolation, and separate calibration of the frame and event responses, including angular sensitivity, gain and shading, crosstalk, and event thresholds.

Despite these advantages, stacked or co-integrated single-chip sensors introduce significant design challenges. Wafer-to-wafer alignment is critical because the photodetection, DVS logic, and digital/system layers must be properly registered; bonding yield and process complexity grow with the number of stacked wafers; thermal coupling can be problematic because high-speed digital activity in the bottom wafer may affect the analog sensitivity and noise of upper layers; inter-layer electrical noise must be managed because event sensing is sensitive to threshold mismatch, leakage, and bias fluctuations; and testability and calibration are harder because behavior depends on interactions among three bonded layers [57,58,61,62]. The DVS path is especially sensitive: since events are triggered by threshold-crossing brightness changes, small perturbations in comparator bias, leakage, substrate noise, or timing skew can alter event rate, polarity balance, or background activity. Vertical integration must therefore be accompanied by careful co-design of analog biasing, supply isolation, timing distribution, shielding, and calibration—a stronger requirement than in conventional frame-only stacked CIS designs because the event path is inherently more sensitive to noise and temporal mismatch [55,57].

From a system perspective, the three-wafer-stacked CIS–DVS architecture suits applications requiring high spatial resolution, high event throughput, low latency, and compact form factor, such as mobile imaging, robotics, AR/VR, automotive perception, and edge-AI vision, where producing dense frames and sparse events from one integrated sensor is highly valuable. At the same time, its process complexity and design cost make it more appropriate for high-value applications than for very low-cost imaging platforms.

### 2.7. Homogeneous-Pixel Computational Hybrid Sensing

A homogeneous-pixel computational hybrid sensing system provides a distinct approach to hybrid image sensing. Unlike pixel-level shared hybrid sensors, which integrate APS frame readout and DVS event readout within the same pixel array using additional event-sensing circuitry, a homogeneous-pixel hybrid sensing system uses a uniform CIS pixel structure across the entire array. Therefore, the term “homogeneous” refers to the use of a uniform pixel structure and conventional CIS-compatible pixel layout. Thus, a homogeneous-pixel hybrid sensing system preserves conventional CIS pixel uniformity, color filter array compatibility, and standard image quality, while generating pseudo-DVS outputs algorithmically. A representative example is the homogeneous hybrid image sensing technique reported by Park et al. [36]. The work demonstrates a hybrid sensing method that produces a conventional 60 fps CIS output and a 1440 fps pseudo-DVS frame output using the same CMOS image sensor platform. Therefore, the architecture can achieve event-like motion information while avoiding the pixel-size penalty associated with dedicated DVS pixels. Park et al. reported a pseudo-DVS pixel pitch of 1.8 μm and demonstrated motion blur-free high-speed image sensing without performance degradation caused by static bad pixels [36].

**Figure 10:**
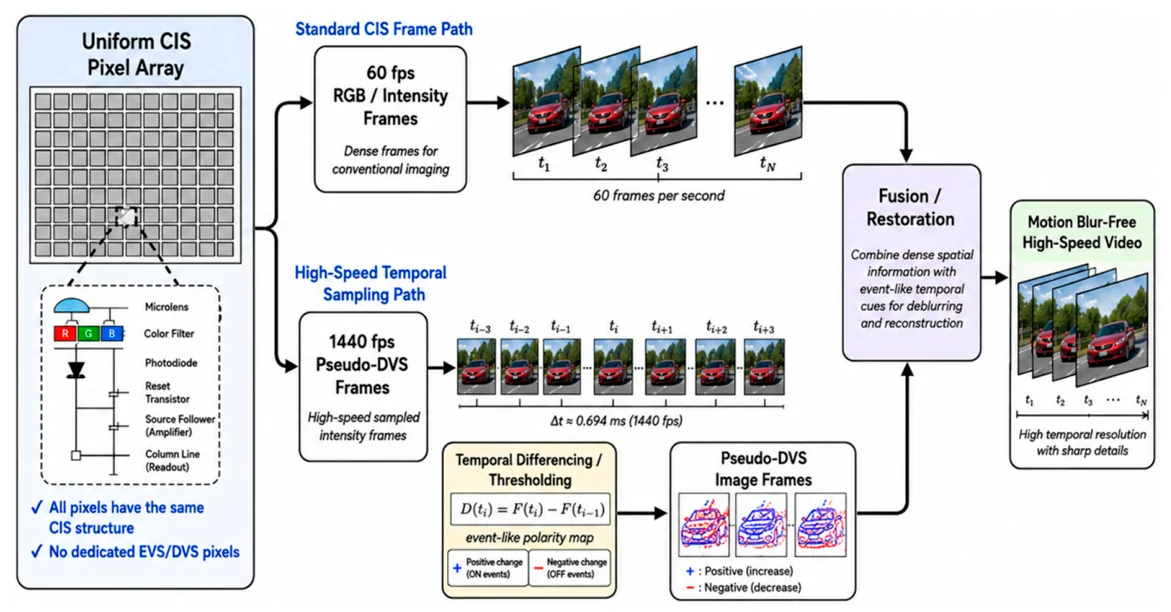
Conceptual structure of a homogeneous-pixel computational hybrid sensing system. A uniform CIS pixel array is used for both conventional frame imaging and pseudo-DVS frame generation. The CIS frame path outputs dense RGB or intensity frames, while the pseudo-DVS path extracts high-speed temporal-difference information from the same pixels. Since no dedicated DVS/EVS pixels are inserted into the array, the architecture preserves spatial uniformity, avoids event-pixel-induced static defects, and maintains compatibility with conventional CIS scaling and image-processing pipelines. Original schematic created by the authors.

Figure 10 illustrates the concept of a homogeneous-pixel computational hybrid sensing system. A single uniform CIS pixel array is operated to generate two types of outputs. The first is a conventional CIS frame output, which provides dense RGB or intensity frames at a standard frame rate. The second is a pseudo-DVS frame output, which extracts high-speed temporal-difference information from the same pixel array. Instead of producing asynchronous address-events directly from dedicated DVS pixels, the pseudo-DVS path generates event-like image frames by comparing successive high-speed samples or by detecting temporal changes in the frame domain. This allows the sensor to provide both human-interpretable image frames and motion-sensitive temporal information while preserving a homogeneous-pixel layout.

The operating principle can be described as frame-domain temporal contrast extraction. Let *F*(*t_i_*) be the image frame or sub-frame sampled at time *t_i_*. A pseudo-DVS signal can be generated by comparing two temporally adjacent samples, for example,

*D*(*t_i_*) = *F*(*t_i_*) − *F*(*t_i_*_−1_) (5)

or by applying a threshold to the temporal difference,

*E*(*t_i_*) = +1, *F*(*t_i_*) − *F*(*t_i_*_−1_) ≥ *T_ON_* –1, *F*(*t_i_*) − *F*(*t_i_*_−1_) ≤ –*T_OFF_* 0, otherwise(6)

Here, *D*(*t_i_*) represents a pseudo-DVS difference image, and *E*(*t_i_*) represents an event-like polarity map. This formulation is conceptually similar to DVS temporal contrast sensing, but the implementation is frame-domain and uses homogeneous CIS pixels. The pseudo-DVS output can therefore be treated as a dense or semi-dense event-like image frame rather than an asynchronous address-event stream.

The main advantage of this approach is that it avoids the need for dedicated event pixels. Conventional DVS pixels require additional circuits, including logarithmic photoreceptors, temporal differencing circuits, comparators, memory, and event-generation logic. This limits pixel scaling and often results in larger pixel pitch than advanced mobile CIS pixels. A homogeneous-pixel hybrid sensing system, by contrast, can inherit the pixel-scaling advantages of CIS technology. This is particularly important for mobile imaging, where pixel pitch, spatial resolution, optical format, and compatibility with existing ISP pipelines are critical. Another advantage is the absence of event-pixel-induced static artifacts. Therefore, the pseudo-DVS signal can be generated without sacrificing regular image sampling. Park et al. demonstrated that the combination of conventional CIS frames and pseudo-DVS frames can be used to obtain motion blur-free high-speed video, showing the potential of a homogeneous-pixel hybrid sensing system for human-perceptual imaging applications [36]. This direction is also consistent with broader computational photography approaches that use short/long exposure combinations, spatially varying exposure, or high-frame-rate sampling to recover sharp images from motion-blurred observations [63,64,65,66].

From the viewpoint of sensor taxonomy, a homogeneous-pixel hybrid sensing system occupies a separate category from both heterogeneous interleaved sensors and vertically stacked CIS–DVS sensors. Its defining property is not the coexistence of two physical pixel circuits, nor the vertical stacking of separate event and frame circuits. Instead, it relies on a uniform CIS pixel array and generates multiple output representations through readout timing, temporal sampling, and signal processing. This makes the architecture attractive for applications where the goal is to add motion-sensitive event-like information to a conventional image sensor without redesigning the pixel array as a true DVS/EVS device. However, the homogeneous-pixel hybrid sensing system also has limitations. First, pseudo-DVS output is derived from sampled frames or sub-frames, so its temporal resolution is limited by the sensor’s high-speed readout capability. A true DVS pixel can generate events asynchronously with microsecond-level latency, whereas a pseudo-DVS frame is usually generated at discrete sampling intervals. Second, because pseudo-DVS generation relies on frame-domain differences, the output may be more sensitive to frame readout noise, quantization, rolling-shutter timing, and exposure settings. Third, the pseudo-DVS output may not fully reproduce the logarithmic response and sparse asynchronous behavior of a physical DVS. For high-speed video reconstruction, the pseudo-DVS sequence should provide enough temporal samples to guide interpolation or restoration. A homogeneous-pixel hybrid sensing system can also benefit from advanced CIS readout techniques. Multiple sampling, burst readout, sub-frame readout, region-of-interest readout, pixel binning, and multi-exposure operation can be used to increase temporal sampling or improve signal-to-noise ratio. Recent CMOS image sensors have explored multiple sampling and high-frame-rate readout to improve image quality and temporal performance [35,67,68,69,70]. These techniques can support a homogeneous-pixel hybrid sensing system because the same pixel array can be operated in different temporal modes to produce both conventional frames and motion-sensitive pseudo-DVS information.

### 2.8. Event-Only Computational Video Reconstruction

Event-only computational video reconstruction represents another important category of hybrid image sensing. This architecture uses only DVS/EVS pixels as the physical sensing front end. No frame-based RGB or grayscale image sensor is included in the hardware. Instead, grayscale video frames are computationally reconstructed from asynchronous event streams using model-based optimization, deep neural networks, or model-aided learning. Because each event reports a local change in log-intensity rather than the intensity itself, the reconstruction of grayscale images or videos from events is an ill-posed inverse problem: the event stream contains rich temporal contrast information, but the absolute intensity baseline, low-frequency texture, and static scene content are not directly observed. Algorithmic video reconstruction attempts to recover these missing intensity components by combining event-camera imaging models with temporal priors, spatial regularization, recurrent memory, and learned image statistics [49,71,72,73,74,75,76,77,78,79].

The basic event-to-video reconstruction problem can be formulated as follows. A simple event-camera model relates the log-intensity change to the accumulated signed events between two time instants:

*logI*(*t_b_*) − *logI*(*t_a_*) ≈ *CΣp_k_δ*(*x* − *x_k_*, *y* − *y_k_*) (7)

where *C* is the contrast threshold, *p_k_* is the polarity of the *k-th* event, and *δ* () denotes the spatial event impulse. This relationship explains why events can recover temporal brightness changes but cannot uniquely determine the absolute image intensity without additional constraints or priors.

**Figure 11:**
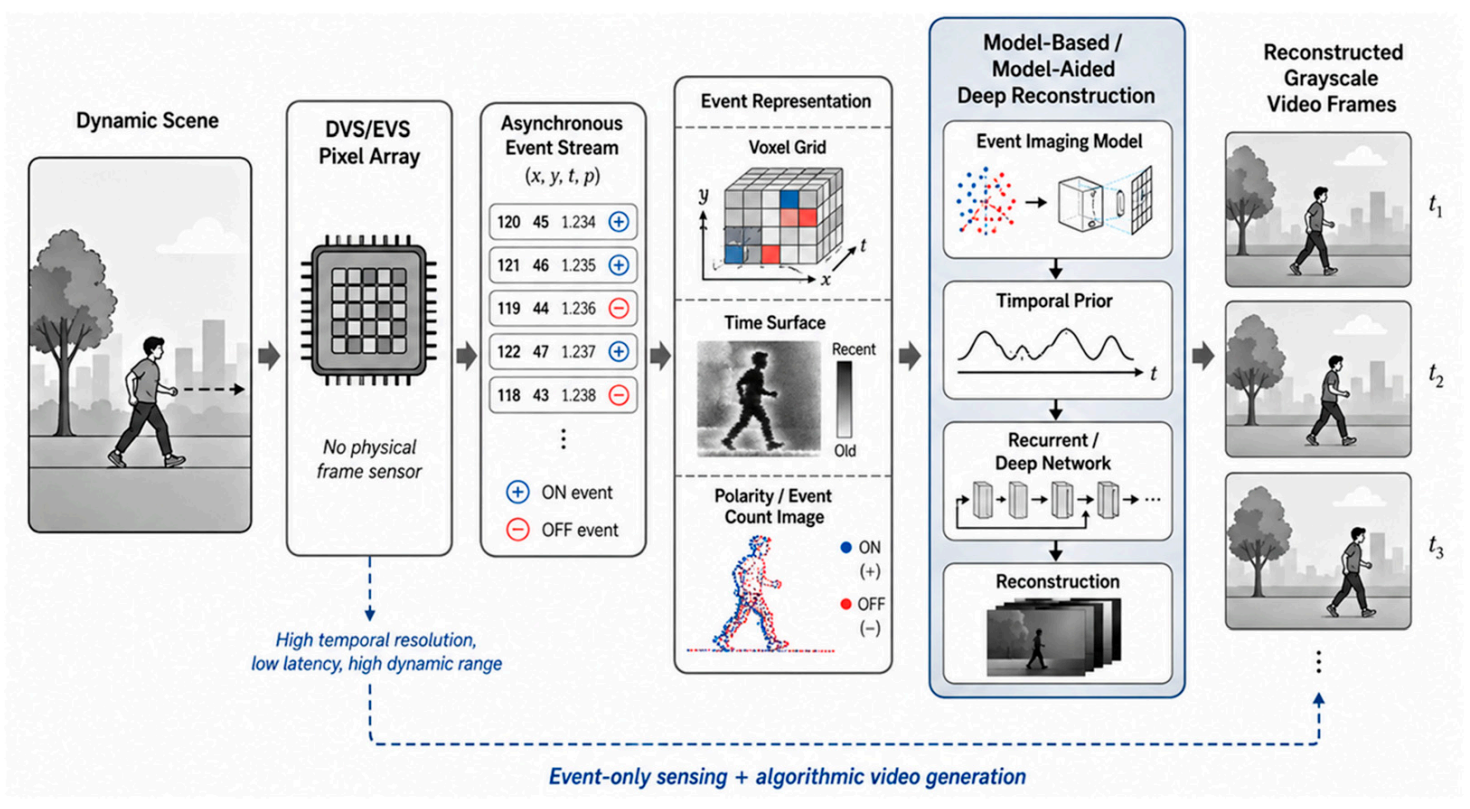
Algorithmic grayscale video reconstruction from an event-only DVS/EVS device. A DVS/EVS pixel array outputs only asynchronous events, without a physical RGB or grayscale frame path. The event stream is converted into an event representation such as a voxel grid, time surface, or polarity tensor. A model-based, learning-based, or model-aided reconstruction algorithm estimates dense grayscale video frames from the event stream. The reconstructed video provides human-interpretable intensity information while preserving the high temporal resolution and high dynamic range advantages of event sensing. Original schematic created by the authors.

Figure 11 illustrates the architecture of an event-only grayscale video reconstruction system. A DVS/EVS pixel array generates asynchronous ON/OFF events from local brightness changes, which are converted into an algorithmic representation such as an event voxel grid, time surface, event-count image, polarity image, or event tensor. A reconstruction algorithm—model-based, learning-based, or model-aided—then estimates grayscale intensity frames, producing a grayscale video sequence that can be displayed to humans or used as input for downstream machine-vision algorithms. Early event-to-image reconstruction methods used model-based optimization. However, purely model-based methods often struggle with noise, texture ambiguity, and complex real-world scenes because the inverse problem is underdetermined [71,72]. Recurrent architectures such as E2VID showed that high-quality intensity video can be reconstructed from events by maintaining temporal memory across event windows [73]. FireNet and related lightweight recurrent models further demonstrated that efficient recurrent networks can reconstruct images from event streams with lower computational cost [49]. Transformer-based methods such as ET-Net introduced long-range spatiotemporal modeling to improve event-based video reconstruction, showing that attention mechanisms can help capture complex temporal dependencies in sparse event data [74]. E2HQV proposes a theory-inspired model-aided deep learning framework for high-quality video generation from event cameras [75]. Instead of relying only on a black-box neural network, E2HQV derives an event-to-video model from event-camera imaging principles and uses this model to guide the deep learning framework. The E2HQV framework highlights a key direction in event-only reconstruction systems: the boundary between sensor hardware and image formation algorithm becomes increasingly blurred. This makes event-only reconstruction fundamentally different from physical CIS–DVS hybrid sensors, but still relevant to hybrid image sensing because it produces both event-domain and frame-like outputs from a single event-based sensing modality.

The main advantage of event-only reconstruction is that it preserves the hardware benefits of DVS/EVS devices. Since no additional frame pixels or RGB readout path is required, the sensor can remain event-only and sparse while providing high temporal resolution. The reconstructed grayscale video can also make event-camera outputs more interpretable to human users and more compatible with conventional computer-vision algorithms. Because events are timestamped asynchronously, reconstructed frames can be generated at user-defined time intervals, enabling high-speed visualization without a high-frame-rate frame sensor. However, event-only reconstruction has limitations: static or low-texture scenes may generate insufficient events, faithful color is unavailable without an additional prior or model, and real-time high-quality reconstruction can require substantial computation, memory, and optimization. Therefore, event-only reconstruction shifts part of the sensing burden from hardware to computation.

Table 2 summarizes the taxonomy of hybrid event–frame architectures from Section 2.3, Section 2.4, Section 2.5, Section 2.6, Section 2.7 and Section 2.8, organized by the level at which frame-like and event-like information are combined. Dual-camera systems combine an RGB and an event camera at the module level—flexible and suitable for research, but suffering parallax, calibration complexity, and larger form factor. Optically aligned systems reduce parallax with shared optics or beam splitters, aiding dense fusion such as deblurring and interpolation, but still need two sensors and extra optics. Pixel-level shared (DAVIS-type) sensors move integration to the focal plane, where APS and DVS readout share the pixel array and optical path, giving intrinsic alignment and compact integration at the cost of pixel-circuit complexity, fill-factor/pitch trade-offs, and limited color—distinct from stacked CIS–DVS sensors, where inserted event pixels create color-fidelity and demosaicing challenges. Cu–Cu-bonded three-wafer-stacked CIS–DVS sensors integrate photodetection, DVS logic, CIS ISP, and DVS readout across three wafers, giving high functional density and compact single-chip implementation but requiring complex bonding, thermal/noise management, and calibration. The last two categories are algorithmically rather than physically hybrid: homogeneous-pixel sensing systems use a uniform CIS arrays and generate pseudo-DVS outputs from high-speed frame differences, preserving uniformity but yielding discrete-time output dependent on readout speed; event-only reconstruction starts from a DVS/EVS device and reconstructs dense grayscale video algorithmically, avoiding a physical frame sensor but requiring substantial computation and being unable to recover absolute intensity or color without additional priors.

The taxonomy therefore contains three distinct higher-level classes. Dual-camera and optically aligned configurations are module-level hybrid vision systems, because physically separate frame and event sensors are combined at the module or optical-path level. Pixel-level shared and stacked CIS–DVS configurations are physically integrated hybrid image sensors, because frame and event sensing functions coexist within a common focal plane or vertically integrated device. Homogeneous-pixel sensing and event-only reconstruction are computationally or functionally hybrid vision systems rather than physically hybrid sensors. The former derives frame- and event-like outputs from a uniform CIS array, whereas the latter reconstructs frame-like intensity data from an event-only front end. They are included in the broader taxonomy because they deliver complementary frame-like and event-like representations, but their hybridization occurs through readout and computation rather than through physical integration of two sensing modalities.

## 3. Sensor Specifications

### 3.1. Latency

Latency is one of the most important sensor specifications for hybrid event–frame sensing systems because it directly determines how quickly a vision system can respond to scene changes. Therefore, latency should not be interpreted only as a sensor data-sheet value. It should be defined as a system-level time delay from a physical scene change to a usable image, event, feature, detection, or control output [80,81,82,83,84,85,86,87,88,89,90,91,92,93]. For a conventional frame-based image sensor, latency is mainly determined by exposure time, frame interval, readout time, ISP processing, memory transfer, and downstream inference. If the frame rate is *f_frame_*, the frame period is

*T_frame_* = 1/*f_frame_* (8)

For a 30-fps camera, *T_frame_* is approximately 33.3 ms, and for a 60-fps camera it is approximately 16.7 ms. A scene change that occurs just after a frame has started may not be available to the processor until the next frame is captured and read out. Therefore, the average temporal waiting time of a frame-based camera is often on the order of *T_frame_*/2, and the worst-case waiting time approaches *T_frame_*, before adding exposure, readout, ISP, and inference delays. The total frame-based latency can be approximated as

*L_frame_* ≈ *T_wait_* + *T_exp_* + *T_read_* + *T_ISP_* + *T_proc_* (9)

where *T_wait_* is the frame sampling delay, *T_exp_* is exposure time, *T_read_* is readout time, *T_ISP_* is image signal processing time, and *T_proc_* is the time required for downstream computer vision or AI processing. This decomposition is useful because it separates sensor-limited latency from system-level processing latency. It also explains why low-latency vision cannot be achieved by increasing frame rate alone; readout, transmission, and algorithmic processing must also be optimized [87,93]. For example, increasing a video stream from 60 fps to 600 fps reduces the frame interval from 16.7 ms to 1.67 ms, but also increases the raw frame data rate by a factor of ten if resolution and bit depth are unchanged. Therefore, frame-rate scaling improves latency at the cost of bandwidth and power consumption. This trade-off is central to hybrid image sensor design, especially when low-latency perception must be implemented in power- or bandwidth-limited systems [87,92,93]. Rolling-shutter distortion has been studied not only as an image-quality problem but also as an inherent timing vulnerability of CMOS image sensors [88]. Recent computational approaches also attempt to restore global-shutter-like video from rolling-shutter inputs, illustrating that temporal skew is both a sensor-design and algorithmic correction problem [89]. Hybrid event–frame sensing systems must therefore consider both inter-frame latency and intra-frame temporal skew.

Event-based sensors have a different latency model. Instead of waiting for the next frame, an event pixel generates an output when the local logarithmic brightness change exceeds a contrast threshold. The latency of an event-based path can be approximated as

*L_event_* ≈ *T_pixel_* + *T_e-read_* + *T_tx_* + *T_e-proc_* (10)

where *T_pixel_* is the pixel response time, *T_e-read_* is event readout time, *T_tx_* is event transmission delay, and *T_e-proc_* is the processing time required to convert events into features, detections, tracks, or control commands. Since event generation is asynchronous and local, event-based sensors can provide much lower temporal latency than frame-based sensors, especially when only a small part of the scene is changing. This property has been exploited in low-latency line tracking, fiducial marker tracking, and high-speed event-camera tracking [80,81,84,85]. However, event-sensor latency is not a single constant. Under low light, photoreceptor response and event reliability may degrade. Therefore, latency should be measured and reported not only at the pixel level, but also at the output-interface and application levels. Low-latency scalable streaming has also become an important system issue because event cameras can generate highly bursty data streams whose transmission delay depends on event activity and interface scheduling [87].

For hybrid event–frame sensing systems, latency can be classified into four levels. Pixel-level latency is the time required for a photodiode, APS pixel, or DVS pixel to respond to optical input. Sensor-output latency is the delay until the frame or event packet is available outside the sensor. Fusion latency includes event accumulation, event-to-frame conversion, temporal alignment, feature fusion, or reconstruction. Task-level latency measures the delay until a usable output, such as a detection, pose estimate, tracking result, or restored frame, is produced. A sensor with low pixel latency may still have high task-level latency if the fusion algorithm or AI model is slow, which is why event-based visual odometry and tracking studies often emphasize asynchronous or lightweight pipelines rather than sensor latency alone [80,84,86].

**Figure 12:**
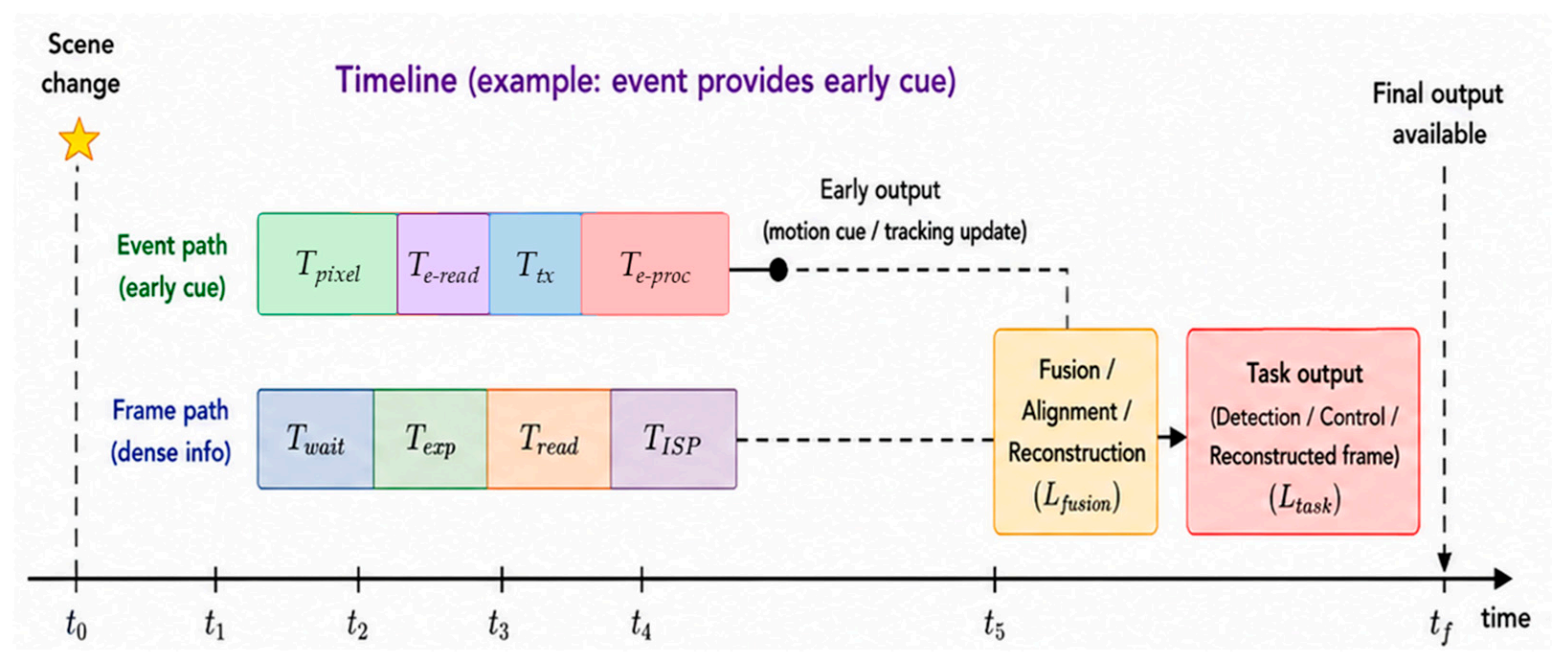
Timeline model for hybrid image sensing. Hybrid latency depends on how frame and event paths are synchronized and fused. The event path can provide early temporal cues, while the frame path provides dense spatial and color information. Original schematic created by the authors.

Figure 12 describes the latency component of hybrid image sensing. In a frame-based path, latency accumulates through exposure, frame waiting, readout, ISP, and AI processing. In an event-based path, local event generation can occur much earlier, but event readout, transmission, and event processing must still be considered. In a hybrid path, the final latency depends on whether the system uses events as low-latency motion cues, as triggers for frame processing, as features for machine vision, or as inputs for image reconstruction. Therefore, the latency advantage of hybrid sensing is maximized when the event path is used directly for early motion, tracking, or detection, rather than being forced into a slow frame-like processing pipeline.

A useful distinction should be made between sensor latency and decision latency. Sensor latency refers to the time required to produce raw frames or events. Decision latency refers to the time required to produce an application-level decision after sensing. For robotics and autonomous systems, decision latency is more important because control commands depend on processed outputs rather than raw sensor packets. Event-based fiducial marker tracking shows that the sparse and asynchronous nature of event streams can support high-rate perception and low-latency control loops when the event-processing algorithm is lightweight [82,83,84]. In a frame-based camera, longer exposure increases photon collection but also increases motion blur. Therefore, latency and blur should be considered together when specifying hybrid event–frame sensing systems for high-speed imaging. In practical inspection and tracking systems, this coupling between temporal sampling, motion blur, and low-latency feature extraction has motivated event-based processing pipelines for automatic visual inspection and high-speed tracking [84,93].

In AR/VR and near-eye sensing, latency is critical because delays in gaze tracking, head motion, or scene updates can reduce user comfort and interaction accuracy. Event-based eye tracking has been explored as a way to achieve very high update rates using asynchronous event streams, while still combining event information with frame-like intensity information for robust pupil estimation [83]. This example illustrates that hybrid sensing can be useful not only for external scene perception but also for human–device interaction, where low latency is essential. Latency specifications should therefore be reported at multiple levels. For frame sensors, useful specifications include frame rate, exposure time, row readout time, global-shutter exposure skew, rolling-shutter scan time, ISP latency, and frame-output delay. Marker tracking, line tracking, event-based visual odometry, and event-streaming studies show that these system-level latency metrics are often more meaningful than nominal sensor response time alone [80,81,82,83,84,85,86,87]. For always-on AIoT, it may correspond to wake-up time after relevant motion is detected. Therefore, latency should be defined from the application endpoint backward to the sensor architecture.

### 3.2. Spatial Resolution

Spatial resolution is a fundamental specification of hybrid event–frame sensing systems because it determines the ability to distinguish fine details, small objects, sharp edges, text, distant targets, and high-frequency textures [94,95,96,97,98,99,100,101,102,103,104,105,106,107,108,109,110,111]. In conventional specifications it is often represented by pixel count or pixel pitch, but for hybrid sensors it should be interpreted more broadly as a system-level property determined by the optical system, pixel sampling, color filter array, sensor modulation transfer function (MTF), event-pixel density, temporal sampling strategy, fusion algorithm, and reconstruction pipeline. The effective spatial resolution of a hybrid sensor is therefore not always identical to the number of physical pixels [94,95,96,97].

For a frame-based image sensor, the sampling-limited resolution is determined by pixel pitch. If the pixel pitch is *p*, the sensor Nyquist frequency can be written as

*f_Nyq_* = 1/2*p* (11)

This equation indicates that smaller pixels increase the maximum spatial frequency that can be sampled without aliasing. For a sensor with active width (*W*) and height (*H*), the number of horizontal and vertical pixels can be approximated as

*N_x_* = *W*/*p*, *N_y_* = *H*/*p* (12)

and the total pixel count is

*N_pix_* = *N_x_N_y_* (13)

Thus, for a fixed optical format, reducing pixel pitch increases pixel count and sampling frequency. This is the main reason why small pixels are widely used in mobile and compact imaging systems. However, reducing pixel pitch also decreases the photon collection area per pixel, full-well capacity, and tolerance to optical and electrical crosstalk. Therefore, spatial resolution must be evaluated together with sensitivity, signal-to-noise ratio, dynamic range, and low-light performance [94,95]. Spatial resolution is more accurately described by MTF, which represents how well contrast is preserved as a function of spatial frequency. The overall system MTF can be approximated as the product of optical, sensor, sampling, color-filter, and processing components:

*MTF_system_*(*f*) = *MTF_optics_*(*f*) · *MTF_sensor_*(*f*) · *MTF_sampling_*(*f*) · *MTF*_*CFA*/*ISP*_(*f*) (14)

This formulation shows that increasing pixel count alone does not guarantee improved effective resolution. Therefore, spatial resolution should be specified using task-relevant measures such as MTF50, limiting resolution, edge spread, line-pair resolution, or detection range, rather than pixel count alone [96,97].

Pixel scaling is especially important in hybrid event–frame sensing systems because architectures allocate spatial sampling resources differently. In dual-camera systems, the frame and event cameras may differ in resolution, pixel pitch, field of view, distortion, and MTF, so fusion resolution is limited by both the higher-resolution sensor and calibration accuracy: projecting events onto an RGB frame makes effective event resolution depend on event-camera pixel pitch, distortion, registration, and interpolation, while projecting RGB into the event-camera coordinate system may downsample it. Optically aligned systems reduce parallax but not resolution mismatch, since the sensors may still differ in pitch, active area, CFA, and spectral response, and beam splitters reduce the photon budget, indirectly affecting resolution through noise and demosaicing [96,97]. Pixel-level shared sensors differ from heterogeneous CIS–DVS pixel-insertion sensors: in a DAVIS-type array APS and DVS sensing are co-located, so the issue is not missing RGB samples but the pixel-area and fill-factor penalty of adding event circuitry, with effective resolution set by pixel pitch, fill factor, photodiode area, and readout layout; intrinsic event–frame registration is gained at the cost of more complex pixels. Stacked CIS–DVS sensors vertically distribute photodetection, event logic, frame readout, and processing, placing more circuitry below the photodetection layer to avoid sacrificing pixel aperture, but still face constraints from photodiode pitch, bonding pitch, interconnect density, optical crosstalk, color-filter alignment, and thermal/noise coupling; stacking improves integration density but does not remove the optical and sampling limits on effective resolution [102,103,104,105,106]. A homogeneous-pixel computational hybrid sensing system provides another strategy. Because all pixels are conventional CIS pixels, the full array can be used for frame imaging, and event-like information is generated through high-speed temporal sampling or temporal differencing. This preserves the spatial uniformity of the image sensor and avoids the missing-sample problem of interleaved APS–DVS arrays. Therefore, homogeneous-pixel hybrid sensing architectures are attractive when high spatial resolution and conventional image quality must be preserved. Event-only algorithmic grayscale reconstruction has the opposite trade-off. Event-based super-resolution methods have shown that higher-resolution intensity images can be reconstructed from lower-resolution event streams by exploiting temporal measurements, motion, and learned priors [98,99,100,101]. However, this is an algorithmic reconstruction process rather than direct sampling by a higher-resolution physical sensor or optical system. Therefore, the output pixel dimensions, the effective reconstructed resolution, and the native physical event-sensor resolution should be reported separately.

Spatial resolution also interacts with color fidelity. In Bayer-patterned CIS sensors, each pixel measures only one-color component, and full-color resolution is recovered by demosaicing. As a result, luminance resolution and chrominance resolution are not identical. Therefore, hybrid sensor design must consider not only total pixel count but also the spatial arrangement of RGB, panchromatic, and event-sensitive pixels. Crosstalk becomes more severe as pixel pitch decreases. Both effects reduce high-frequency contrast and degrade MTF. Microlens design, light guides, deep trench isolation, backside illumination, and optimized photodiode structures are widely used to reduce crosstalk and preserve spatial resolution in small-pixel image sensors [102,103,104,105,106]. These effects are especially important in hybrid sensors because additional circuitry, event logic, or interconnect structures may complicate optical stack design.

**Figure 13:**
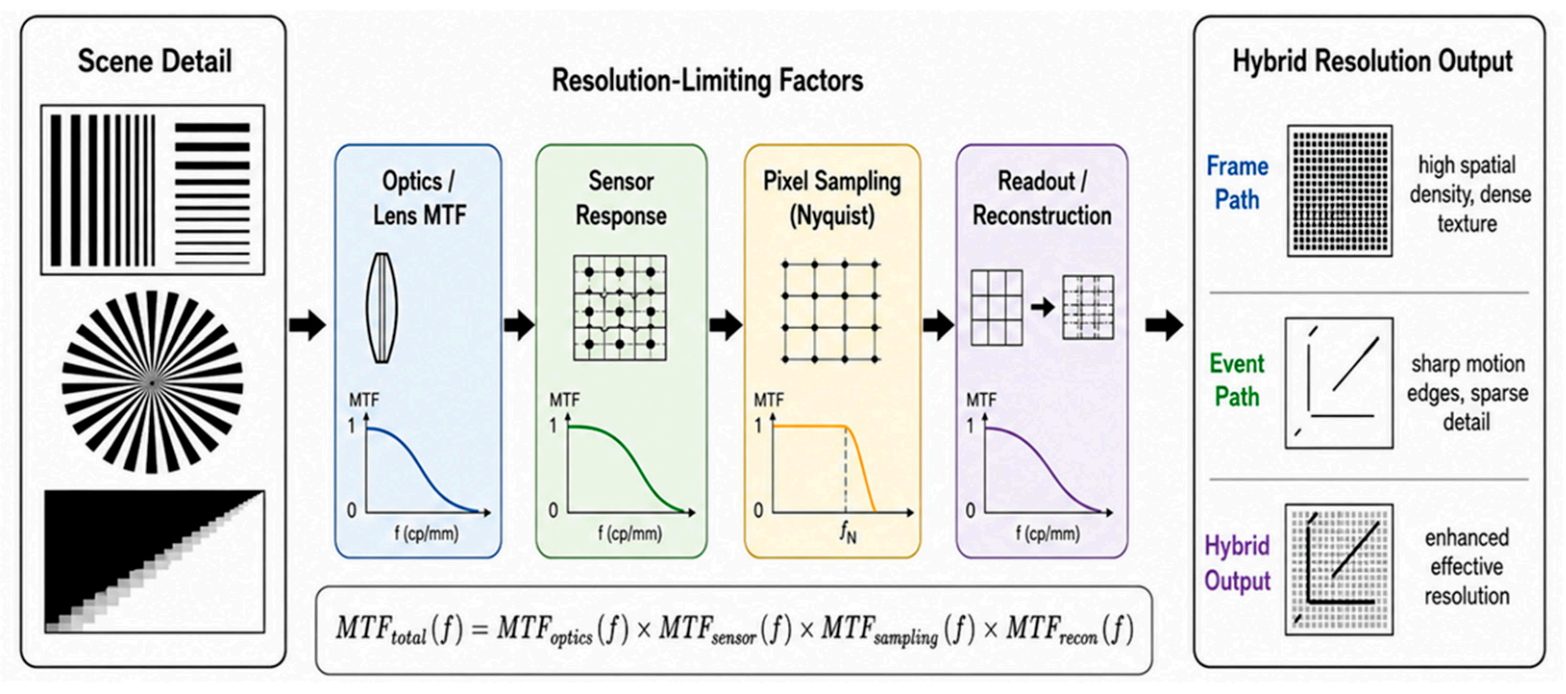
Spatial-resolution budget of hybrid event–frame sensing systems. Physical sensor resolution is determined by the number, pitch, and arrangement of the native frame and event sensing sites. Optical resolution is determined by the spatial contrast delivered to these sites by the lens and optical stack. Algorithmically reconstructed resolution is produced through demosaicing, registration, event–frame fusion, interpolation, or super-resolution and should be distinguished from both physical and optical resolution. The final effective and task-level resolution depends on the complete optical–sensor–algorithm chain and should be validated using spatial-detail measurements rather than output pixel count alone. Original schematic created by the authors.

For hybrid event–frame sensing systems, three resolution concepts should be clearly distinguished. Physical sensor resolution refers to the number, pitch, and spatial arrangement of the actual frame and event sensing sites. It defines the native sampling grid and its associated Nyquist limit, but does not by itself indicate how much spatial detail is resolved. Optical resolution refers to the spatial detail and contrast delivered to the sensor plane by the lens and optical stack and is governed by optical MTF, diffraction, aperture, aberrations, defocus, field curvature, chief-ray angle, and optical crosstalk. Consequently, a high physical pixel count cannot compensate for insufficient optical resolution. Algorithmically reconstructed or super-resolved resolution refers to the spatial detail estimated after demosaicing, denoising, registration, event–frame fusion, interpolation, deblurring, or super-resolution. Its nominal output dimensions may exceed the native frame or event resolution, but a larger output grid alone does not demonstrate additional resolved information. Event- or multi-frame-assisted super-resolution may recover detail from complementary temporal or subpixel measurements; however, such resolution remains reconstruction-dependent and should be reported separately from physical and optical resolution and validated using MTF, edge-response, line-pair, or ground-truth-based measurements [96,97,98,99,102,103,104,105,106]. Fusion resolution and task-level resolution are derived system-level measures: the former describes the spatial scale at which the modalities are registered and combined, whereas the latter describes whether the final system resolves the objects or features required by the application. Accordingly, the final effective resolution is determined by the cascade of optical imaging, pixel sampling, sensor response, event–frame registration, and reconstruction or fusion processing, as shown in Figure 13. A high-resolution frame path provides dense spatial structure and an event path provides edge and motion information with high temporal resolution, but if the event path has much lower spatial resolution, the registration error is large, or fusion is performed at a low-resolution feature level, the full frame resolution may not be useful for the final task. Conversely, if events are used mainly as temporal cues for deblurring or interpolation, lower event spatial resolution may still be sufficient.

### 3.3. Color Fidelity

Color fidelity is a critical specification for hybrid event–frame sensing systems because it determines how accurately the spectral and perceptual color of a scene is reproduced. For human-perceptual imaging it affects naturalness, skin-tone reproduction, display quality, mobile photography, AR/VR rendering, and video consistency; for machine vision it matters when semantic recognition, classification, traffic-sign detection, food inspection, medical imaging, agricultural monitoring, or material recognition relies on color cues. Color fidelity should therefore be treated as a sensor- and system-level specification, not merely an ISP tuning parameter. In a conventional RGB sensor, color combines the spectral response of the optics, CFA, microlens stack, photodiode quantum efficiency, and ISP color correction. For a color channel c ϵ {R, G, B}, the sensor response can be modeled as

*y_c_* = ∫ *E*(*λ*)*R_s_*(*λ*)*T_opt_*(*λ*)*F_c_*(*λ*)*Q*(*λ*) *dλ* + *n_c_* (15)

where *E*(*λ*) is the illuminant spectrum, *R_s_*(*λ*) is the scene surface reflectance, *T_opt_*(*λ*) is the optical transmittance, *F_c_*(*λ*) is the spectral transmittance of the color filter for channel *c*, *Q*(*λ*) is the photodiode quantum efficiency, and *n_c_* is noise. This equation shows that color fidelity depends on both the scene spectrum and the camera spectral sensitivity. Therefore, two cameras with different spectral sensitivities can produce different RGB values for the same object under the same illumination [112,113,114,115,116,117,118]. The goal of color characterization is to transform camera-dependent RGB responses into a device-independent color representation, usually CIE XYZ or a standard RGB color space. A common linear model is

*M_CCM_* [*R_wb_*, *G_wb_*, *B_wb_*]*^T^* (16)

where *M_CCM_* is a color correction matrix and *R_wb_*, *G_wb_*, *B_wb_* are white-balanced sensor responses. ISO 17321-1 defines procedures for digital camera color characterization, while CIE colorimetry provides the colorimetric basis for mapping physical spectra to human-perceptual color coordinates [112,113]. Perceptual camera characterization methods further show that minimizing error in perceptual color spaces can improve visual color reproduction compared with least-squares fitting in XYZ alone [114,115]. Therefore, color correction matrices are approximations, and color errors depend on the illuminant, object spectrum, and calibration set [115,116,117,118,119]. Color fidelity is also coupled to noise. Therefore, color filter design is a trade-off among colorimetric accuracy, sensitivity, spatial reconstruction quality, and noise robustness. Studies on optimal spectral sensitivity functions and CFA design show that color filter spectra affect both color reproduction and spatial reconstruction quality [119,120,121]. More recent work has also considered joint design of camera spectral sensitivity and color correction under noise constraints, indicating that color fidelity and noise performance should be co-optimized rather than treated independently [122].

**Figure 14:**
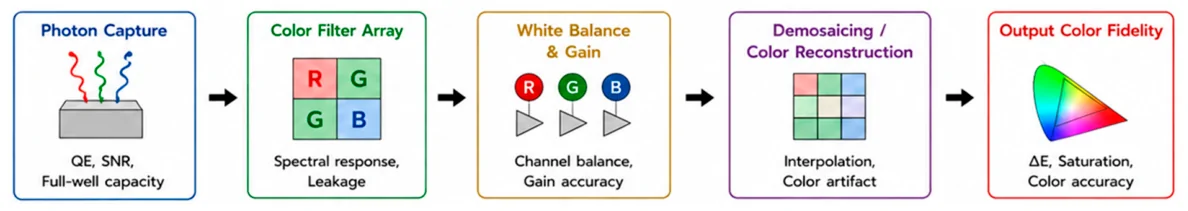
Color-fidelity budget of hybrid event–frame sensing systems. Color fidelity is determined by the scene spectrum, illumination, optical transmittance, color filter array, photodiode quantum efficiency, crosstalk, white balance, color correction matrix, demosaicing, ISP, and event–frame fusion. The RGB frame path provides measured color information, whereas the event path usually provides brightness-change information. In hybrid event–frame sensing systems, the fusion algorithm should improve temporal or dynamic-range performance without degrading colorimetric accuracy, chroma resolution, or temporal color consistency. Original schematic created by the authors.

In hybrid event–frame sensing, the color-fidelity problem depends strongly on the architecture. In dual-camera systems, the RGB camera provides physical color, whereas the event camera provides brightness-change events; RGB fidelity can stay high with a conventional sensor and ISP, but fusion may introduce color artifacts if events are misregistered, temporally misaligned, or applied unevenly across channels, so fidelity depends on geometric calibration, synchronization, and fusion strategy. In optically aligned systems, sharing a common axis reduces parallax, but beam splitters or prisms can alter spectral balance and reduce the photon budget; wavelength-dependent transmission or reflection gives the RGB sensor a spectrally modified scene requiring additional calibration, so both spatial alignment and spectral transmission should be characterized. Pixel-level shared sensors differ again: many DAVIS implementations output monochrome APS intensity rather than RGB, useful for algorithm development, SLAM, tracking, and visual–inertial perception but less suitable for color-critical use; color DAVIS variants are possible, but color filters reduce photon efficiency and complicate the dense APS–DVS pixel, so the limitation relates to pixel-circuit complexity, fill factor, sensitivity, and color-filter integration. Stacked CIS–DVS sensors can better preserve color if the CIS path keeps a conventional RGB pixel structure and ISP while event logic is integrated in separate layers, though stacking can still affect color through optical-stack thickness, metal routing, inter-layer shading, wafer alignment, thermal effects, and crosstalk; crosstalk-correction methods for CFA sensors show optical and electrical crosstalk cause both desaturation and spatial blur, making crosstalk a color-fidelity as well as a spatial-resolution issue [60]. A homogeneous-pixel computational hybrid sensing system is advantageous for color fidelity because all pixels can remain conventional CIS pixels. Since no dedicated DVS/EVS pixels are inserted into the array, the regular CFA layout can be preserved, and conventional demosaicing and ISP pipelines can be used. Therefore, this architecture is attractive for applications that require high color fidelity and motion-sensitive temporal information at the same time. Event-only reconstruction systems represent the most difficult case for color fidelity. A standard DVS/EVS measures local brightness changes, not absolute intensity or color. Therefore, an event-only reconstruction system can produce frame-like intensity video but cannot guarantee faithful color reproduction without additional information. Color event cameras, color-filtered event sensors, periodic RGB keyframes, active colored illumination, or learned color priors may be used to synthesize or reconstruct color information, but such outputs should be distinguished from directly measured color [123,124,125,126,127,128]. The Color Event Camera Dataset demonstrates that color event cameras and color event simulation can support continuous-time HDR color video reconstruction, but the color information still depends on sensor spectral design and reconstruction algorithms [123]. Illumination-based color reconstruction from DVS data further illustrates that color can be inferred under controlled active illumination, but this is not equivalent to passive full-color capture by a conventional RGB sensor [124]. Figure 14 illustrates the color-fidelity budget of hybrid event–frame sensing systems. The color pipeline begins with the scene spectrum and illumination, passes through optics and color filters, is converted into sensor RGB responses, and is then processed by white balance, color correction, demosaicing, tone mapping, and event–frame fusion. Therefore, color-fidelity evaluation should include both raw sensor characterization and final fused-output evaluation.

Accordingly, hybrid-ISP evaluation should not rely solely on PSNR or SSIM. Colorimetric assessment should include metrics such as ΔE_00_ and chroma resolution, while video assessment should characterize temporal color stability, white-balance transitions, and color flicker under changing or mixed illumination.

### 3.4. Power Consumption

Power consumption is a central specification for hybrid event–frame sensing systems because it determines whether the sensor can be used in mobile devices, AR/VR headsets, drones, robotics, always-on AIoT nodes, automotive perception modules, and battery-powered surveillance systems. In conventional image sensors, power consumption is often reported as a sensor-level value under a specific resolution, frame rate, bit depth, and supply voltage. However, for hybrid event–frame sensing systems, this definition is insufficient. A hybrid image sensor contains or emulates both frame-like and event-like sensing paths, and its total energy cost includes pixel operation, analog readout, column-parallel ADCs, event arbitration, timestamping, digital control, ISP, event–frame fusion, memory traffic, interface transmission, and downstream AI processing. Therefore, power consumption should be treated as a system-level specification rather than a single sensor data-sheet number.

The total power of a hybrid vision system can be approximately decomposed as

*P_frame_* + *P_event_* + *P_fusion_* + *P*_*memory*/*interface*_ + *P_AI_* (17)

where *P_frame_* represents the power of the frame-based imaging path, *P_event_* represents the power of the event-based path, *P_fusion_* represents event–frame alignment, reconstruction, or feature fusion power, *P_memory_*_/*interface*_ represents data movement and off-chip communication power, and *P_AI_* represents downstream neural-network or task-processing power. This decomposition is useful because the lowest-power hybrid architecture is not necessarily the one with the lowest pixel power. A sensor that slightly increases on-chip circuit complexity may reduce total system power if it substantially reduces frame bandwidth, memory traffic, or AI workload.

For a frame-based image sensor, power consumption increases with pixel count, frame rate, bit depth, ADC activity, and output bandwidth. A simplified proportional model can be written as

*P_frame_* ∝ *N_pix_* · *f_frame_* · *B* · *E_read_* (18)

where *N_pix_* is the number of pixels, *f_frame_* is the frame rate, *B* is the bit depth, and *E_read_* is the energy required to read and digitize one pixel sample. This relationship explains why high-resolution, high-frame-rate RGB imaging is expensive in terms of power. Increasing frame rate to reduce latency or motion blur directly increases the number of pixel samples and ADC conversions per second. Therefore, for power-constrained hybrid event–frame sensing systems, it is important to avoid unnecessary dense frame readout when the scene is static or when only motion information is required. Readout circuits and ADCs are major contributors to CIS power. Timing optimization of readout circuits and reference DAC operation can reduce power in high-frame-rate image sensing systems without sacrificing image quality [129]. Similarly, adaptive or hybrid ADC architectures can reduce dynamic power by changing resolution or operation mode according to illumination or required precision [130]. Resolution scaling is another effective way to reduce frame-path power. Kim et al. demonstrated a CIS that supports multiple resolution modes from full resolution down to 1/64 resolution, showing that scaled-resolution imaging can reduce total power when the scene is in a low-activity or monitoring state [131]. This principle is directly relevant to hybrid sensors: the event path can provide low-cost temporal activity information, while the frame path can be activated at full resolution only when dense spatial or color information is needed.

Event-based sensing has a different power model. A DVS/EVS does not output full frames at a fixed rate; instead, it outputs events only when local brightness changes exceed a threshold. Therefore, the event-path power can be approximated as

*P_event_* ≈ *P_bias_* + *R_event_* · *E_event_* (19)

where *P_bias_* is the static bias power required to operate the pixel and peripheral circuits, *R_event_* is the event rate, and *E_event_* is the energy required to generate, arbitrate, timestamp, transmit, and process one event. This equation highlights the activity-dependent nature of event sensing. When the scene is static, *R_event_* can be low, and the event path may consume much less data-processing and transmission power than a frame camera. When the scene is highly dynamic, contains flickering illumination, or uses a low contrast threshold, *R_event_* can increase significantly, and the event path may no longer be low power. The power advantage of event cameras is therefore not guaranteed by the sensor principle alone. It depends on scene activity, bias settings, threshold tuning, readout architecture, and the efficiency of downstream event processing. Event-driven smart vision sensors and neuromorphic processing pipelines have shown that event sparsity can be exploited for low-power surveillance, tracking, and object recognition, but the algorithms must be designed to operate directly on sparse asynchronous data rather than converting all events into dense frames [132,133,134,135]. If the event stream is first accumulated into high-rate dense frames and then processed by a conventional CNN, much of the potential power advantage may be lost.

Hybrid event–frame sensing systems should therefore be designed around energy proportionality, consuming power roughly in proportion to useful scene activity and task difficulty. Ideally a low-power event or activity path stays always on while the high-power RGB frame path, ISP, memory interface, and AI accelerator activate only when needed—for example, a surveillance sensor monitoring in event-dominant mode, switching to low-resolution frames on weak activity, and enabling full-resolution imaging and inference only when a relevant target appears—which is more efficient than running all paths at full resolution and frame rate. The optimal strategy depends on architecture. Dual-camera systems give RGB and event paths independent power domains so the RGB path can be duty-cycled while the event camera stays active, but two sensors, two optical paths, and synchronization hardware raise baseline power. Optically aligned systems may share optics but still need separate readout and interfaces, gaining efficiency mainly from improved fusion, while beam splitters reduce photon efficiency and may force longer exposure or higher gain. Pixel-level shared sensors reduce module power by integrating frame and event sensing in one focal-plane device, but pixel-level integration raises circuit complexity, so power must account for APS readout, DVS readout, bias current, arbitration, timestamping, and output activity. Stacked CIS–DVS sensors give the most compact integration and on-chip processing that cuts off-chip transfer, but packing active circuits into a small volume makes thermal management, leakage, and power-domain design critical, so power should be reported with operating mode, event rate, frame rate, temperature, and output bandwidth. Homogeneous-pixel sensing systems need no extra DVS pixel circuit but generate pseudo-DVS information by high-speed sampling, raising internal readout bandwidth and digital power unless localized, reduced-resolution, or compressed readout is used. Event-only reconstruction systems minimize sensor complexity but shift power to computation, since grayscale reconstruction may need recurrent or transformer models, voxel-grid formation, and memory access, so they should not be called low-power solely because the sensor is event-based.

**Figure 15:**
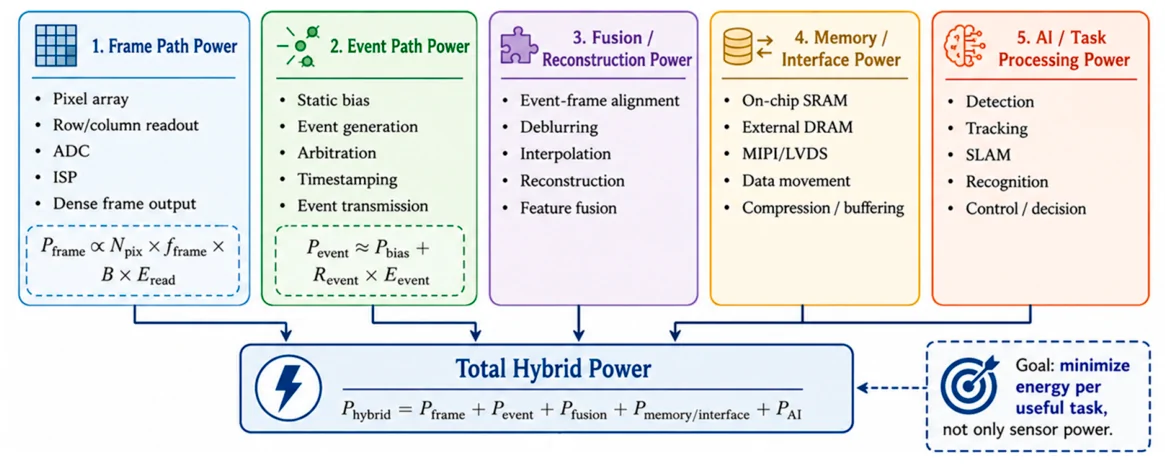
Power-consumption budget of hybrid event–frame sensing systems. Total hybrid event–frame system power comprises frame-path power, event-path power, fusion or reconstruction power, memory and interface power, and downstream AI-processing power. The frame path scales mainly with pixel count, frame rate, bit depth, and ADC activity, whereas the event path scales with static bias and event rate. A power-efficient hybrid sensor uses event or activity information to reduce unnecessary dense frame readout, memory transfer, and AI processing. Original schematic created by the authors.

Figure 15 illustrates the power-consumption budget of hybrid event–frame sensing systems. The frame path consumes power through exposure control, row/column readout, ADC conversion, ISP, and dense frame transfer [136,137,138]. The event path consumes static bias power and activity-dependent event generation, arbitration, timestamping, and transmission power. Fusion, memory, and AI processing then determine the final system-level power. The key design goal is not simply to minimize sensor power, but to minimize energy per useful perception output under the target application workload.

### 3.5. Form Factor

Form factor is a critical specification for hybrid event–frame sensing systems because hybrid sensing often requires additional pixels, circuits, optical paths, readout channels, memory, or processing blocks compared with a single-modality sensor. In mobile devices, AR/VR headsets, drones, robotics, automotive modules, wearables, endoscopes, and always-on AIoT cameras, the allowable module volume is limited, so form factor should be defined not only by die size but by the complete imaging module, including optics, package, mechanical housing, interface routing, thermal path, and calibration stability. It can be described at three levels: the sensor-level form factor (die area, pixel pitch, peripheral circuit area, stacked layers, bonding pitch, package footprint); the camera-module-level form factor (number of sensors and lenses, beam splitters, prism blocks, lens barrel, optical total track length, module height); and the system-level form factor (flexible printed circuit routing, processor placement, heat spreading, mechanical rigidity, calibration tolerance, and product integration). This multi-level definition is necessary because a compact die does not automatically yield a compact module if the optical path or calibration structure is large. The optical system often dominates form factor. In a conventional camera module, the total track length is constrained by focal length, field of view, aperture, sensor size, and image-quality requirements. If the same field of view and F-number must be maintained, simply shrinking the sensor does not always reduce the whole module thickness because the lens still needs sufficient optical path length and correction elements. Wafer-level camera technologies address this issue by fabricating and aligning optics, spacers, and image sensors at the wafer level, enabling smaller and lower-cost camera modules for high-volume applications [139,140]. Wafer-level optics and wafer-level packaging are therefore important technologies for compact hybrid image sensors, especially when hybrid sensing must be integrated into mobile or wearable products.

Dual-camera event–frame systems are least compact, requiring two sensors, two lens assemblies, two packages, and a baseline that adds parallax and calibration burden, so they suit research and automotive test platforms rather than ultra-compact products. Optically aligned systems share one lens through a beam splitter or prism, reducing parallax but adding optical-module volume for the combiner and alignment. Pixel-level shared sensors integrate APS and DVS readout into one focal-plane device sharing one lens, approaching a single-sensor module footprint at the cost of pixel-circuit complexity that limits scaling, fill factor, and color. Stacked hybrid sensors are among the most attractive, distributing photodetection, readout, event logic, memory, ISP, and processing vertically to reduce lateral area without enlarging the pixel-plane footprint, enabled by three-dimensional wafer-level stacked BSI technologies [141]; however, stacking increases fabrication and bonding complexity, thermal density, and stack height, which in mobile and AR/VR systems can be more restrictive than footprint, and digital-layer heat affects dark current, noise, and event-threshold stability, so stack thickness, package height, and thermal design should be reported. Homogeneous-pixel sensing system keeps a conventional CIS array and optical module with no dedicated DVS pixels or second sensor, preserving a near-standard form factor, though the high-speed readout and processing may add peripheral area and power. Event-only reconstruction systems use only a DVS/EVS device, so the front end is compact, but human-interpretable high-quality color video may need additional optics, illumination, processing, or memory, shifting form factor from sensor to computation.

**Figure 16:**
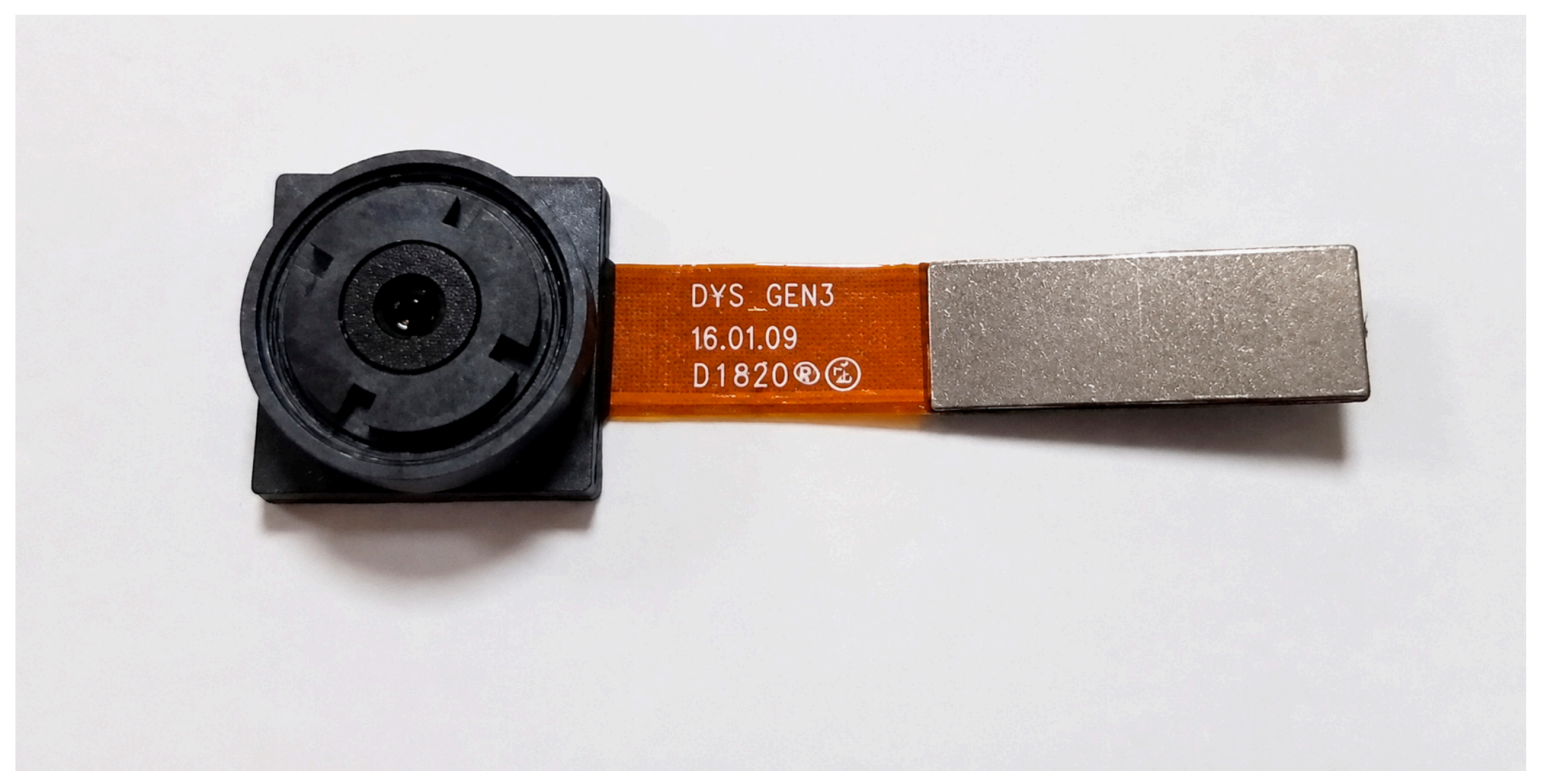
A representative DVS camera module. The image illustrates the external appearance and module-level implementation of a DVS device; it does not provide a scale-normalized comparison of sensor die area, package footprint, optical track length, complete system volume, power consumption, or computational hardware. The final form factor depends on the complete optical, sensing, interface, thermal, and processing configuration. The photograph was taken by the authors.

Computational imaging offers another route to compact form factor. Lensless and coded-aperture cameras replace bulky lens assemblies with thin masks, diffusers, or computational elements near the sensor: FlatCam demonstrated a thin bare-sensor camera using a coded mask and computational reconstruction [142], and DiffuserCam demonstrated lensless single-exposure 3D imaging with a diffuser in front of a standard sensor [143]. These are not hybrid event–frame sensors, but they show that form-factor reduction often shifts complexity from optics to computation, a principle hybrid sensors can follow via stacked sensing, on-chip processing, or algorithmic reconstruction. Compact optics also matter: two-photon direct laser writing has produced ultracompact multi-lens objectives for endoscopy, micro-robotics, and wearable imaging [144], and wafer-level meta-aspheric lenses have been explored for ultracompact wide-FOV near-infrared cameras targeting smartphones and AR glasses [145,146,147,148], relevant because the optical module often limits miniaturization more than the sensor die. Form factor also affects calibration: with two optical paths, mechanical drift, thermal expansion, vibration, and assembly tolerance can change event–frame alignment, whereas a compact monolithic or stacked architecture improves calibration stability, while dual-camera and beam-splitter systems may need periodic recalibration. Interface routing is another aspect: outputting dense RGB frames, sparse events, pseudo-DVS frames, reconstructed video, metadata, timestamps, or AI features requires more pads, lanes, serializers, and package routing, which can bottleneck compact modules, so on-chip compression, event filtering, feature extraction, and shared event–frame interfaces should be considered from the start. Figure 16 presents a photograph of a representative DVS module used in sensing research and applications. This photograph illustrates the external module-level implementation and the degree of visible hardware integration, but it should not be interpreted as a direct or scale-normalized comparison of complete system form factor. The total form factor of a hybrid event–frame system is determined not only by the camera or sensor module itself but also by the complete optical–sensor–package–processor chain, including the number of optical paths, lens assemblies, beam splitters, calibration structures, interface electronics, thermal components, and downstream processing hardware. Therefore, a compact module photograph does not necessarily indicate a compact complete system.

## 4. Sensor Configuration and Challenges

### 4.1. Sensor Configuration

Sensor configuration is the process of selecting the physical and functional arrangement of frame-like and event-like sensing paths according to application requirements. As discussed in Section 3, hybrid event–frame sensing specifications include latency, spatial resolution, color fidelity, power consumption, and form factor. These specifications are strongly coupled. Therefore, the optimal configuration cannot be determined from one specification alone. Instead, the sensor configuration should be derived from the relative priority of the target application.

In this review, sensor configuration refers to four design choices. The physical configuration determines whether the system uses separate sensors, shared optics, shared pixels, stacked wafers, homogeneous CIS pixels, or event-only hardware. The modality configuration determines whether the output includes RGB frames, grayscale frames, pseudo-DVS frames, asynchronous events, reconstructed frames, or task-level features. The fusion configuration determines whether event and frame information are fused at the data, feature, decision, or reconstruction level. The operating configuration determines frame rate, event threshold, resolution mode, duty cycle, ROI mode, and on-chip and off-chip processing split. A useful framework begins by identifying the primary information required: faithful color and dense spatial texture require a physical RGB frame path; very low latency, high-speed motion cues, or robustness to motion blur require an event or event-like path; stringent form-factor constraints favor a single-chip or homogeneous-pixel configuration over a dual-camera system; and if algorithmic reconstruction is acceptable and physical color is not required, an event-only configuration can be considered.

The first configuration class is the dual-camera event–frame system, in which a conventional RGB or grayscale frame camera and an event camera are mounted together. This configuration is attractive when flexibility, modular prototyping, sensor replacement, and independent optimization of the two modalities are important. It is also useful when the application requires high-quality frame data and high-quality event data without compromising either pixel design. For example, stereo hybrid event–frame cameras have been proposed for 3D perception by using separate high-quality event and frame cameras rather than relying on tightly coupled pixel-level hybrid sensors [149]. The limitation is that dual-camera systems require extrinsic calibration, synchronization, larger module volume, and compensation for parallax. Therefore, this configuration is most appropriate for robotics platforms, autonomous-driving research, laboratory systems, and applications where module size is less constrained.

The second class is the optically aligned event–frame system, where RGB/frame and event sensors share a common optical axis through a beam splitter, prism, or coaxial optical path. This configuration should be selected when pixel-level event–frame correspondence is more important than form factor. Because parallax is reduced, optically aligned systems are suitable for event-guided deblurring, frame interpolation, HDR reconstruction, photometric stereo, and dense event–frame fusion. Event Fusion Photometric Stereo Network (EFPS-Net), for example, demonstrates that RGB and event cameras can be combined to estimate surface normals under ambient illumination, illustrating the usefulness of event–frame configuration for tasks in which the frame path provides intensity observations and the event path provides temporally resolved illumination or motion cues [150]. However, optically aligned systems increase optical complexity and may reduce photon efficiency because light must be split between sensing paths.

The third class is the pixel-level shared sensor, in which APS frame readout and DVS event readout are integrated within the same pixel array. This configuration is suitable when form factor and intrinsic event–frame spatial alignment are required. Since frames and events originate from the same focal-plane reference, pixel-level shared sensors avoid the parallax and extrinsic calibration burden of dual-camera systems. However, this advantage is obtained by increasing pixel-circuit complexity, which can reduce fill factor, limit pixel scaling, and constrain color capability. Therefore, this configuration is appropriate when accurate spatiotemporal alignment is more important than maximum RGB image quality or independent optimization of the two modalities.

The fourth class is the stacked CIS–DVS single-chip sensor, in which photodetection, frame readout, event logic, memory, ISP, or event-processing circuits are vertically integrated. This configuration is appropriate when small form factor, high integration density, and synchronized event–frame output are required simultaneously. It is particularly attractive for mobile, automotive, robotics, and AR/VR applications where dual-camera modules are too bulky but both dense frames and low-latency event information are valuable. The main trade-offs are fabrication complexity, wafer bonding, thermal density, and calibration of multiple signal paths.

The fifth class is the homogeneous-pixel hybrid sensing system, in which all pixels remain conventional CIS pixels and event-like information is generated through high-speed temporal sampling or temporal differencing. This configuration should be selected when conventional image quality, regular CFA layout, small pixel pitch, and existing camera-module compatibility are more important than true asynchronous event generation. It is particularly attractive for mobile imaging and motion-blur-free high-speed video because it preserves a uniform CIS pixel array while generating pseudo-DVS information. However, the pseudo-event path is limited by frame-domain sampling and high-speed readout, so it is not equivalent to a true DVS/EVS path.

The sixth class is the event-only algorithmic reconstruction configuration, in which the physical sensor is a DVS/EVS and frame-like grayscale video is reconstructed algorithmically. This configuration is suitable when the hardware must be compact, sparse, and low-latency, and when grayscale or reconstructed intensity output is acceptable. It is less suitable when faithful color, guaranteed static-scene texture, or radiometric accuracy is required. Therefore, event-only reconstruction should be selected for event-centric machine perception, compact sensing, high-speed visualization, or applications where learned reconstruction is acceptable.

After selecting the physical architecture, the next decision is the fusion level. Data-level fusion combines raw frames, event images, voxel grids, time surfaces, or event-count maps. It is useful for image restoration, deblurring, interpolation, and reconstruction, but it can be computationally expensive and sensitive to calibration errors. Feature-level fusion extracts features from each modality and combines them in a neural network or signal-processing pipeline. RGB–event collision prediction with self-attention, for example, uses separate RGB and event encoder branches followed by attention-based fusion, showing that feature-level fusion can improve robustness but may increase memory and FLOPs [151]. Decision-level fusion combines outputs such as detections, tracks, or classifications from separate modality-specific pipelines. Reconstruction-level fusion generates a restored image, HDR video, grayscale video, or high-frame-rate sequence from frame and event inputs. The choice of fusion level should match the sensor configuration. Dual-camera and optically aligned systems often use data- or feature-level fusion because both modalities are physically available. Pixel-level shared and stacked sensors can support tighter sensor-level or readout-level fusion because event and frame signals are more closely aligned. The best operating mode depends on whether the application is limited by latency, resolution, color fidelity, power, or form factor.

For autonomous driving and robotics, the configuration should prioritize low latency, motion robustness, and stable perception under fast scene changes. RGB–event moving-object detection shows that event streams can complement RGB frames by providing temporal motion information for dynamic objects, especially when frame-based methods are limited by motion blur or adverse conditions [152]. In such applications, a dual-camera, optically aligned, or stacked CIS–DVS configuration can be selected depending on module-size constraints. If the goal is experimental flexibility, a dual-camera configuration is appropriate. If small form factor and calibration stability are required, stacked or integrated configurations are preferable.

For object recognition and action recognition, the configuration should balance RGB texture information and event temporal information. SSTFormer, for example, combines a memory-support Transformer for RGB frames with a spiking neural network for raw event streams and a multimodal bottleneck fusion module [153]. This suggests that recognition-oriented hybrid sensors should preserve both dense RGB features and temporally sparse event information. Therefore, event–frame feature-level fusion is often more suitable than raw data-level fusion for recognition tasks.

For semantic segmentation and scene understanding, dense spatial context from frames remains important, but event streams can improve robustness under motion, illumination variation, or sparse activity. Recent event-image fusion networks for semantic segmentation use modality-adaptive refinement, attention, or gated fusion to combine dense image information with event-based temporal cues [154,155,156,157,158]. These examples suggest that for segmentation-oriented hybrid sensors, the configuration should preserve sufficient frame resolution while ensuring accurate event–frame synchronization and event representation quality.

For UAV collision prediction and obstacle avoidance, latency and power may dominate over color fidelity. In this case, the sensor configuration may use a low-frame-rate RGB path together with an event path, or even an event-dominant configuration if task accuracy is sufficient. RGB–event collision prediction studies show that fusion can improve prediction performance for difficult cases, but the added fusion network can increase computation [151]. Therefore, for embedded UAV systems, the configuration should be selected not only by accuracy but also by memory, FLOPs, and energy per decision.

For industrial inspection and photometric measurement, radiometric stability, spatial resolution, and calibration may dominate. Event–frame photometric stereo illustrates that an event camera can complement RGB imaging under ambient illumination by providing temporal information that conventional RGB-only photometric stereo lacks [150]. In such cases, optically aligned configurations may be preferable because accurate event–frame registration is more important than minimum form factor. However, if the system must be miniaturized, stacked or single-chip hybrid configurations may become more attractive.

**Figure 17:**
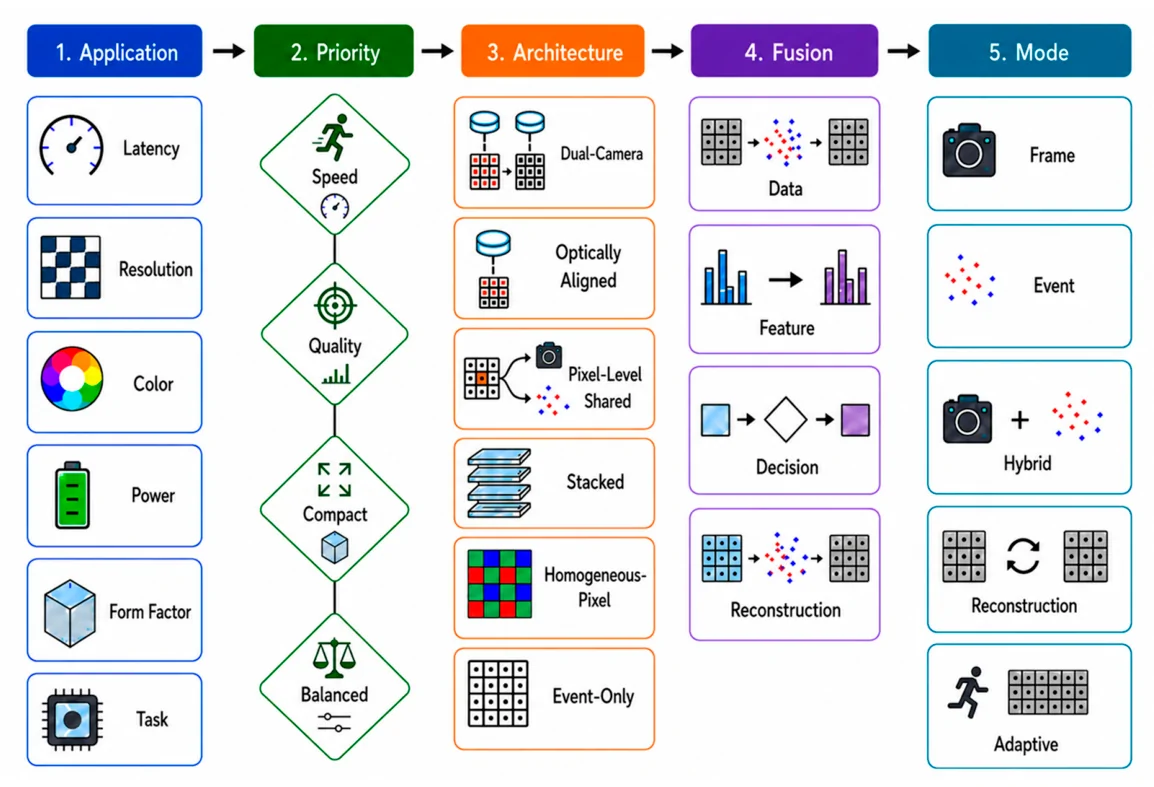
Architecture-selection flow for hybrid event–frame sensing. The hybrid event–frame architecture should be selected by mapping application requirements to physical architecture, output modality, fusion level, and operating mode. Color-critical applications require a physical RGB frame path. Low-latency applications require event or event-like sensing. Compact applications favor stacked, shared, homogeneous-pixel, or event-only configurations. Calibration-sensitive reconstruction tasks favor optically aligned configurations. Power-constrained applications require event-driven operation and lightweight fusion. Original schematic created by the authors.

Figure 17 presents an architecture-selection flow for hybrid event–frame sensing. The flow begins with the dominant application requirement. If color fidelity and dense texture are mandatory, a physical RGB frame path should be preserved. If latency and motion robustness dominate, an event or event-like path should be included. If form factor is the strongest constraint, integrated, stacked, homogeneous, or event-only configurations should be considered. If calibration accuracy is more important than form factor, optically aligned configurations are preferable. Finally, if computation or power is limited, the fusion level should be selected carefully to avoid unnecessary dense reconstruction or heavy neural processing.

**Figure 18:**
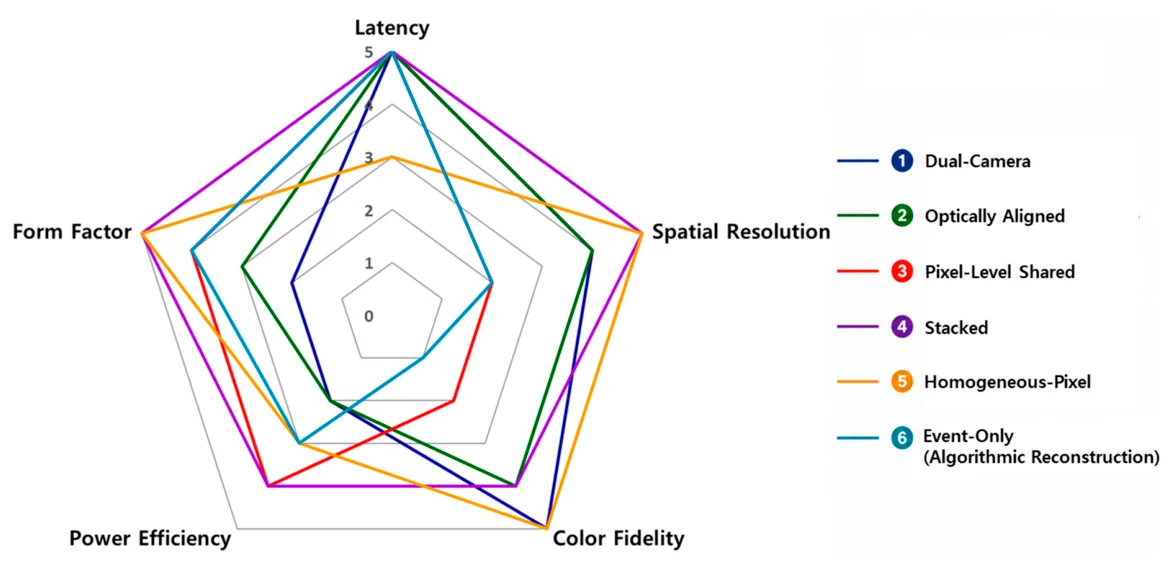
Evidence-supported ordinal trade-off radar chart for representative hybrid event–frame sensing architectures and systems. Scores follow the five-level rubric defined in the text and are anchored to representative values reported in the cited literature. Higher scores indicate shorter native sensing latency, higher effective spatial resolution, better physical color fidelity, greater power efficiency, or a more compact form factor. No weighted sum, mean score, or radar-polygon area is used to rank the architectures. Original schematic created by the authors.

Figure 18 uses a five-level ordinal scoring rubric rather than a direct linear normalization of heterogeneous measurement results. Each score is anchored to representative quantitative data reported in the cited literature, and higher scores indicate more favorable performance. For latency, the score refers to the native response or temporal-sampling latency of the event or event-like sensing path: a score of 5 corresponds to ≤0.1 ms, 4 to >0.1–0.5 ms, 3 to >0.5–2 ms, 2 to >2–10 ms, and 1 to >10 ms. Architectures retaining a physical DVS/EVS path therefore generally receive a score of 5 because representative sensors report response latencies of several to several tens of microseconds, whereas homogeneous-pixel pseudo-DVS sensing is limited by high-speed CIS readout and temporal differencing and exhibits approximately 1 ms latency, resulting in a score of 3 [5,36,37,38].

For spatial resolution, the base score is determined from the native physically sampled dense-frame resolution: 5 corresponds to ≥10 Mpixels, 4 to 2–<10 Mpixels, 3 to 0.5–<2 Mpixels, 2 to 0.05–<0.5 Mpixels, and 1 to <0.05 Mpixels or the absence of a physically sampled dense frame. One score level is deducted when the usable fusion resolution is substantially limited by severe event–frame resolution mismatch, a frame-to-event pixel-pitch ratio of approximately 4 or greater, parallax or registration error, or reconstruction-only output. For example, representative stacked hybrid sensors provide 15-Mpixel or 35.6-Mpixel RGB paths together with approximately 1-Mpixel event paths, whereas early pixel-level shared sensors commonly provide sub-megapixel frame and event resolution [7,23,55,56].

For color fidelity, the score is based on the fraction of spatial sites that directly acquire physical RGB color information and on the amount of required spectral or computational correction. A score of 5 corresponds to 100% conventional CFA coverage without missing color samples; 4 corresponds to ≥75–<100% physical color-sample coverage or a complete RGB path requiring path-specific spectral calibration; 3 corresponds to 25–<75% direct color coverage or substantial color reconstruction; 2 corresponds to >0–<25% direct color sampling or architectures in which color is available only in limited variants; and 1 corresponds to 0% physically acquired color information. Thus, conventional RGB dual-camera and homogeneous-pixel systems can receive a score of 5, whereas a HybridEVS pattern containing 14 color pixels and 2 event pixels in a 4 × 4 block has 87.5% direct color-sample coverage and is assigned a base score of 4. An event-only reconstruction system receives a score of 1 because it does not directly measure scene color.

For power efficiency, the score is based on the reported active power of the complete sensing front end, including the sensor, required readout circuits, and mandatory on-sensor or near-sensor processing when such data are available: 5 corresponds to ≤100 mW, 4 to >100–600 mW, 3 to >600 mW–1 W, 2 to >1–2 W, and 1 to >2 W. Partial-mode values, such as DVS-only power, are identified separately and are not treated as complete hybrid-system power. For example, the representative stacked and homogeneous implementations summarized in Table 4 report approximately 525 mW and 845 mW, respectively, corresponding to scores of 4 and 3 under this rubric, whereas a reported 64 mW DVS-only operating mode is treated only as a partial-mode reference [23,36,55,56]. When complete system power is unavailable, the score is estimated from the reported sensor and processing components.

For form factor, the score is determined using the number of sensor packages, lens modules, additional optical elements, and mandatory external processing components because complete module dimensions are not consistently reported. A score of 5 corresponds to one sensor package and one lens without a beam splitter or mandatory external reconstruction processor; 4 corresponds to one sensor and one lens with an additional processing package or increased package volume; 3 corresponds to two sensors sharing one lens through a beam splitter or prism; 2 corresponds to two independently packaged sensors with two lens modules; and 1 corresponds to systems requiring more than two sensing or optical modules or a bench-top alignment structure. Accordingly, stacked and homogeneous-pixel hybrid sensing architectures generally receive the highest form factor scores, optically aligned systems receive an intermediate score, and dual-camera systems receive a lower score.

Dual-camera event–frame systems provide strong sensing flexibility because the RGB/frame camera and event camera can be independently selected and optimized. This allows high-quality RGB imaging and high-quality event sensing to coexist without pixel-level compromise. Such configurations are useful in robotics, autonomous-driving research, and 3D perception platforms, including stereo hybrid event–frame camera systems [149]. However, they generally have lower compactness and power efficiency because they require two sensors, two optical modules, synchronization, calibration, and additional data interfaces. Optically aligned event–frame systems improve spatial correspondence by sharing a common optical axis or using a beam splitter. This makes them suitable for reconstruction-sensitive tasks such as deblurring, interpolation, photometric stereo, and dense event–frame fusion. For example, event–frame photometric stereo demonstrates the benefit of combining frame intensity information with event-domain temporal cues for surface-normal estimation [150]. Nevertheless, optically aligned systems remain relatively bulky because of the beam splitter or prism, and their optical efficiency can be reduced by light splitting. Pixel-level shared sensors improve form factor and spatial alignment by integrating APS and DVS functions into one focal-plane sensor. Their strongest advantage is intrinsic event–frame registration, because frame and event outputs share the same optical reference. Instead, their main trade-offs are increased pixel-circuit complexity, reduced fill factor, larger pixel pitch, limited color capability, and more complex dual-mode readout. Therefore, in the radar chart, pixel-level shared sensors should be scored highly in latency and power efficiency, moderately in form factor and spatial resolution, and lower in color fidelity compared with conventional RGB-preserving architectures. Stacked CIS–DVS sensors provide a balanced configuration. By vertically integrating photodetection, frame readout, event logic, memory, and digital processing, stacked architectures can maintain a compact footprint while supporting both frame and event outputs. This makes them attractive for embedded vision, mobile imaging, AR/VR, robotics, and automotive applications where form factor and synchronized multimodal sensing are both important. The main limitations are process complexity, wafer bonding, thermal density, and calibration of multiple signal paths. Therefore, the radar chart shows stacked CIS–DVS sensors as a strong all-around option rather than an architecture that maximizes only one criterion. A homogeneous-pixel hybrid sensing system preserves the conventional CIS pixel array and generates pseudo-DVS outputs through high-speed temporal sampling or temporal differencing. This configuration is advantageous when conventional image quality, regular CFA sampling, and existing camera-module compatibility are important. Since no dedicated DVS pixels are inserted into the pixel array, color fidelity and spatial uniformity can be preserved. However, the event-like output is discrete-time and depends on high-speed readout, so its latency and power efficiency may not match those of a true asynchronous DVS path. For this reason, the radar chart represents homogeneous-pixel hybrid sensing as a compact and color-preserving architecture with balanced but application-dependent latency and power characteristics. Event-only algorithmic reconstruction systems minimize sensing hardware because they use only DVS/EVS pixels and reconstruct grayscale video or frame-like output computationally. Because no physical RGB or grayscale frame sensor is present, color fidelity and static-scene texture reconstruction are limited. Event-only reconstruction also shifts complexity from sensor hardware to algorithms and processors, as shown in event-to-video and event-based reconstruction studies [49,51,159]. Therefore, Figure 18 shows this architecture as highly compact and power-efficient, but weaker in color fidelity and direct spatial image quality.

Overall, Figure 18 highlights that hybrid event–frame architecture selection is a trade-off problem rather than a simple ranking. Architectures with the best sensing quality are not necessarily the most compact or energy-efficient. Conversely, compact architectures may require compromises in color fidelity, spatial sampling, or algorithmic complexity. Therefore, the appropriate configuration should be selected by matching the dominant application requirement to the architecture. If calibration flexibility and maximum modality quality are required, dual-camera systems are suitable. If dense pixel-level fusion is required, optically aligned systems are preferred. If form factor is critical, shared, stacked, homogeneous-pixel hybrid sensing, or event-only reconstruction configurations should be considered. If balanced performance across sensing quality, form factor, and integration is required, stacked CIS–DVS or homogeneous-pixel hybrid sensing architectures are especially attractive.

### 4.2. Open Challenges

**Figure 19:**
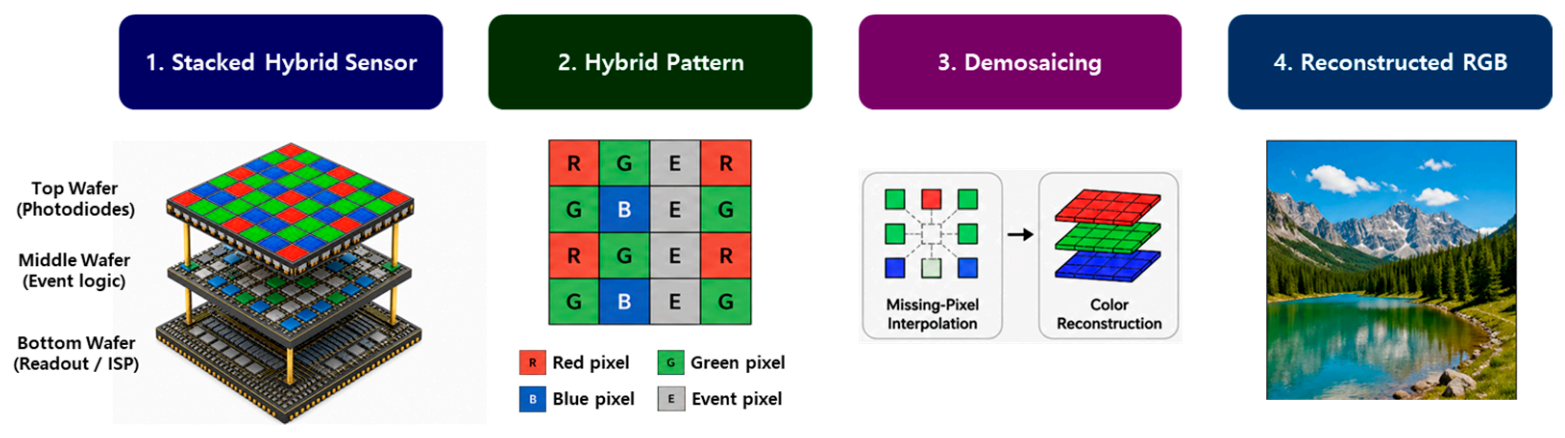
Open challenges in HybridEVS color-fidelity design. Stacked or integrated HybridEVS arrays can provide compact event–frame sensing, but event pixels inserted into the RGB or Quad Bayer array create missing color samples and defect-like raw data. Dedicated HybridEVS demosaicing is required to reconstruct high-quality RGB images while preserving event-pixel density. Original schematic created by the authors.

Hybrid event–frame sensing has progressed rapidly from loosely coupled event–frame systems to compact single-chip and stacked implementations. Among the configurations in Section 4.1, stacked hybrid sensors are especially competitive, combining high integration density, compact form factor, synchronized event–frame readout, and possible on-chip ISP or event signal processing by vertically distributing photodetection, event logic, memory, readout, and digital processing. However, they still face open challenges including color fidelity, pixel-layout optimization, event-pixel density, demosaicing, readout bandwidth, power density, calibration, thermal stability, event-threshold mismatch, and manufacturability. Color fidelity is among the most important challenges when event pixels are inserted into an RGB or Quad Bayer array, because some positions sense events rather than color, leaving missing or non-color pixels that conventional Bayer or Quad Bayer demosaicing cannot directly handle. This is illustrated by the MIPI 2024 Challenge on Demosaic for HybridEVS Camera: the reported HybridEVS pattern combines a Quad Bayer color filter array with event pixels (Figure 19), and the task is to reconstruct high-quality RGB images from HybridEVS raw data containing event and defect pixels [160]. Because event pixels capture no color or texture, they behave like structured missing samples, making HybridEVS demosaicing harder than conventional demosaicing. The challenge shows this is not only a hardware issue but also an ISP and computational imaging issue: it provided training, validation, and test data and evaluated submissions using PSNR (Peak Signal-to-Noise Ratio) and SSIM (Structural Similarity Index Measure) [160], with top methods achieving high quality, indicating that learned demosaicing can substantially compensate for the missing color [160,161], though the ISP pipeline must be specifically designed for the hybrid raw pattern.

DemosaicFormer [161] adopts a coarse-to-fine demosaicing framework consisting of a coarse demosaicing network and a pixel-correction network. The coarse stage produces an initial RGB estimate from HybridEVS raw data, while the correction stage mitigates artifacts caused by event pixels and defective pixels. This suggests that high-quality HybridEVS demosaicing requires both local interpolation and global contextual reasoning. Another MIPI 2024 method, Event Camera Demosaicing via Swin Transformer and Pixel-focus Loss, treats event-camera demosaicing as a missing-pixel RAW-domain reconstruction problem [162]. Therefore, loss functions that emphasize difficult pixels may be useful for maintaining perceptual image quality. The MIPI 2024 challenge report also summarizes additional team solutions, including Multi-Resolution SwinMaxIR for QCFA Raw Demosaic, Two-Stage Joint Inpainting and Demosaicing Network, Step-by-step De-mosaic Model for Hybrid EVS Camera, Efficient and Explicit Hierarchies Modelling Network for HybridEVS Camera Demosaic, and Multi-Stage Fusion Demosaicing with Integrated Pixel Attention and Residual Learning [160]. This differs from conventional demosaicing, where every pixel usually contains a valid color sample.

Recent studies following the MIPI 2024 challenge further reinforce this direction. TSANet separates event-pixel inpainting and Quad Bayer demosaicing using a two-stage network with state-space augmented cross-attention, improving performance while reducing parameters and computation compared with DemosaicFormer [163]. BMTNet introduces a binarized Mamba–Transformer architecture for lightweight Quad Bayer HybridEVS demosaicing, targeting edge-device deployment [164]. These works show that the next challenge is not only reconstruction accuracy but also efficient deployment. RGB–event ISP is another emerging research direction. Instead of treating event data only as an auxiliary input for post-processing, RGB–Event ISP studies how event information can be integrated into the image signal processing pipeline itself [165]. EvRAW similarly shows that event-guided structural and color modeling can be useful for RAW-to-sRGB reconstruction [166].

The central design question is how many event pixels can be inserted into the RGB array without unacceptable image-quality loss [167,168,169,170,171,172,173,174,175,176,177,178,179,180,181,182,183,184,185,186,187,188,189]. An excessively high RGB-to-DVS ratio may provide insufficient event-pixel density, whereas an excessively low ratio reduces the number of available color samples and complicates demosaicing. The HybridEVS pattern used in the MIPI 2024 challenge contains two event pixels and fourteen color pixels in a 4 × 4 block, corresponding to an RGB-to-DVS ratio of approximately 7:1 and an event-pixel occupancy of 12.5%. Since all seven final teams achieved PSNR values above 40 dB and the top method reached 44.8464 dB with an SSIM of 0.9854, the approximately 7:1 RGB-to-DVS ratio was shown to support high pixel-wise reconstruction fidelity and structural similarity when a dedicated learned demosaicing pipeline was used [160,161]. However, these results do not by themselves establish colorimetric accuracy, chroma resolution, or temporal color stability, because the challenge evaluation was primarily based on PSNR and SSIM. This proves feasibility under the given dataset, pattern, metric, and algorithms, not that 7:1 is universally optimal, since the acceptable ratio also depends on color-fidelity requirements, model complexity, power, bandwidth, thermal limits, and real-time constraints. Ratios such as 15:1 and 31:1 correspond to lower event-pixel occupancies of approximately 6.25% and 3.125%, respectively, as shown in Table 3. In this review, these ratios are presented only as illustrative design hypotheses for exploring the trade-off between event density and preservation of physical color samples. They are not experimentally validated recommendations. A 31:1 ratio represents a low event-density, image-quality-oriented hypothesis, whereas 15:1 represents an intermediate hypothesis between 31:1 and the published 7:1 MIPI configuration. Their actual suitability remains application- and implementation-dependent. Establishing a practical or optimal RGB-to-DVS ratio requires a controlled ratio-sweep study in which sensor format, CFA design, event-pixel placement, event threshold, optical crosstalk, noise, demosaicing architecture, training data, runtime, power, and thermal conditions are held consistent. Evaluation should include color difference, chroma resolution, spatial detail, temporal color stability, event-task accuracy, latency, and energy consumption.

Several challenges remain. Although PSNR and SSIM are useful for measuring pixel-wise reconstruction fidelity and structural similarity, they do not directly quantify colorimetric accuracy or temporal color artifacts. Future HybridEVS and hybrid-ISP benchmarks should therefore include CIE color-difference metrics, such as CIEDE2000 ΔE_00_ measured using calibrated color targets and natural scenes, together with chroma-resolution measures such as chroma MTF or color-edge response. Video evaluation should additionally characterize temporal color stability under static, changing, and mixed illuminants by measuring frame-to-frame color differences, chroma fluctuations, white-balance transition behavior, and color flicker. These evaluations should be performed for both the standalone RGB reconstruction and the final event–frame fused output, because event-guided processing may improve temporal sharpness while still introducing spatial or temporal color inconsistencies. Perceptual quality and downstream machine-vision accuracy should also be reported as complementary metrics.

Table 4 compares representative CIS–DVS hybrid image sensor technologies. The comparison focuses on the CIS resolution ratio, CIS and DVS pixel pitch, and reported power consumption, because these parameters directly affect spatial image quality, event-sensing granularity, and mobile/edge-AI applicability. The comparison indicates that stacked heterogeneous CIS–DVS sensors can provide high event throughput with compact integration and low power, whereas homogeneous pseudo-DVS sensing can minimize the effective event-pixel pitch and avoid static image degradation caused by dedicated DVS pixels.

## 5. Discussion

Hybrid event–frame sensing is not limited to the combination of a conventional RGB camera and an event camera. It represents a broader design direction in which dense spatial/color sensing, sparse temporal sensing, sensor readout, image signal processing, and edge-AI computation are jointly optimized. The taxonomy reviewed in this paper shows that hybrid sensing can be implemented at different integration levels: camera-module level, optical-path level, focal-plane level, wafer-stack level, homogeneous-pixel hybrid sensing level, or algorithmic reconstruction level [190,191,192,193,194,195,196,197,198]. Therefore, ‘hybrid event–frame sensing’ is used here as the broader system-level concept, whereas ‘physically integrated hybrid image sensor’ is reserved for devices that incorporate both frame and event sensing functions within a common focal plane, chip, or wafer stack. Recent surveys of event-based vision also support this system-level view, because event-camera research has expanded from sensor hardware to reconstruction, restoration, recognition, tracking, depth estimation, datasets, and task-level benchmarks [190,191,197].

The most important implication of this review is that there is no universally optimal hybrid sensor configuration. Dual-camera systems provide flexibility and independent optimization of each modality, but they suffer from form-factor, calibration, and synchronization limitations. Optically aligned systems improve event–frame correspondence but increase optical complexity. Pixel-level shared sensors reduce module volume but trade off pixel-circuit complexity, fill-factor reduction, pixel-pitch constraints, dual-mode readout complexity, and limited color capability in many implementations. Stacked CIS–DVS sensors provide strong overall competitiveness because they can integrate frame and event functions in a compact single-chip form. A homogeneous-pixel hybrid sensing system preserves conventional CIS image quality while generating pseudo-DVS information from high-speed temporal sampling. Event-only reconstruction systems minimize sensing hardware but shift the burden to computational reconstruction. This diversity of configurations is consistent with recent event–frame task studies, where different applications such as interpolation, deblurring, tracking, pedestrian detection, and synthetic-aperture imaging adopt different combinations of frames, events, and fusion algorithms [199,200,201,202,203,204].

Among these configurations, stacked hybrid sensors are especially attractive for future embedded and mobile vision, because photodetection, event logic, frame readout, ISP, memory, and event processing can be vertically integrated in a compact footprint, suiting applications that need both high-quality RGB frames and low-latency temporal information. They also reveal a central challenge: the sensor must preserve conventional CIS image quality while adding event sensing. In particular, inserting event pixels into an RGB or Quad Bayer array disrupts the sampling pattern, making color fidelity an ISP- and algorithm-level challenge [160,161,162], and the acceptable RGB-to-DVS ratio depends on the strength, cost, and robustness of the demosaicing pipeline. This points to a broader conclusion: future hybrid sensors will increasingly rely on computational sensor design. Whereas conventional sensors optimize the pixel array and ISP around a known CFA and dense frame readout, hybrid raw data may contain RGB pixels, event pixels, defect pixels, pseudo-DVS frames, asynchronous event streams, timestamps, and calibration metadata, so the “raw image” becomes a multimodal measurement and the reconstruction, demosaicing, event representation, and neural ISP must be considered part of sensor design. Deep-learning surveys for event-based vision show event representations and architectures strongly affect reconstruction, restoration, recognition, and 3D performance [190,191], and event-to-video, event-guided deblurring, frame interpolation, and autofocus studies show output quality depends on the interaction of events, frames, temporal modeling, and reconstruction [200,201,203].

A major unresolved issue is how hybrid sensors should be evaluated. Recent benchmark and challenge studies in event-based vision have started to compare accuracy, model complexity, latency, robustness, and task performance, but a unified benchmark for hybrid event–frame sensing hardware and systems is still lacking [192,193,194,195]. Event-based eye-tracking challenges and large-scale action-recognition datasets illustrate the growing need for task-specific evaluation beyond conventional image-quality metrics [192,193,194]. Therefore, datasets should include realistic HybridEVS raw patterns, event-pixel noise, RGB-pixel noise, defect pixels, optical crosstalk, color shading, temperature effects, and event–frame synchronization metadata. The TUMTraf Event dataset shows that RGB–event fusion can support traffic perception and roadside sensing, while also highlighting the challenges of calibration, synchronization, and domain-specific fusion [196]. Sim-to-real studies for event cameras further show that synthetic training data and real sensor behavior can differ substantially, which is an important issue for HybridEVS ISP and sensor-algorithm co-design [205,206]. Edge AI is another key issue, because heavy off-chip fusion can eliminate the latency and power benefits of hybrid sensing. Therefore, future hybrid event–frame systems should support lightweight, near-sensor, or on-chip processing. Event-based detection, tracking, and mixed frame- and event-driven pedestrian-detection studies suggest that energy-efficient hybrid perception requires algorithms that exploit event sparsity rather than converting all event data into dense frame representations [202,204,205]. Calibration remains another open problem. For event–frame 3D perception and stereo vision, calibration and synchronization directly affect depth accuracy and task reliability. Recent RGB–event roadside datasets and event-based stereo surveys emphasize that calibration, synchronization, and dataset realism remain major research gaps for event-based 3D perception [196,197,198]. This suggests that hybrid sensor design should include calibration hardware and metadata generation as first-class design requirements.

Another important question is whether hybrid event–frame systems should prioritize human-perceptual imaging or machine vision. A human-facing camera needs color fidelity, spatial detail, low artifacts, and temporal consistency, whereas a machine-vision sensor may prioritize latency, power, feature stability, and task accuracy over perceptual quality. Hybrid event–frame systems can serve both, but a single output pipeline may not be optimal for both, so future sensors may provide multiple representations: high-quality RGB frames for viewing, event streams for low-latency perception, pseudo-DVS frames for motion processing, and compact features for AI accelerators. This multi-output design is supported by the diversity of recent event–frame tasks, including frame interpolation, deblurring, tracking, pedestrian detection, motion segmentation, and event-guided reconstruction [199,200,201,202,203,204,205,206,207,208,209,210]. Architecture should also be selected by deployment scenario: dual-camera systems for research and algorithm development; optically aligned systems for dense restoration; homogeneous-pixel hybrid sensing systems and stacked sensors for compact consumer devices; event-only or event-dominant configurations for event-centric robotics and low-power sensing; and stacked CIS–DVS sensors for high-end embedded vision, provided color fidelity, thermal management, and ISP complexity are solved. This requirement-driven selection is consistent with the broader literature, where reconstruction, recognition, tracking, segmentation, and 3D perception each favor different event representations and fusion strategies [190,191,197,202,203,204,205].

Recent frame-based computer-vision studies also provide useful algorithmic references for future hybrid sensing pipelines. Jiang et al. developed a real-time poultry-monitoring system that combines an attention-enhanced YOLO detector with BoT-SORT for multi-object trajectory tracking [211]. This example shows the importance of combining discriminative spatial features with efficient temporal association in practical tracking applications. Zhang et al. proposed a Multi-Scale Temporal Difference Unit for cross-view gait recognition, explicitly computing temporal differences over multiple frame intervals to emphasize dynamic motion cues while suppressing static appearance information [212]. Although these methods operate on conventional frame or silhouette sequences rather than physical event streams, their attention, temporal association, and multi-scale difference mechanisms are relevant to hybrid event–frame vision. In a hybrid implementation, dense RGB frames could provide appearance and semantic information, whereas event streams or pseudo-DVS outputs could provide low-latency motion cues for association, trajectory updating, and dynamic-feature extraction. Such extensions should nevertheless be experimentally validated, because event data differ from conventional frame differences in temporal sampling, noise characteristics, polarity representation, and sparsity.

Table 5 summarizes representative measured application-level outcomes for the architecture classes discussed in this review. Each row corresponds to a specific published implementation rather than an average value assigned to the entire architecture class. We report only values explicitly provided by the original study, including sensor-output or end-to-end latency, task accuracy, reconstruction quality, dynamic range, power, calibration performance, model size, FLOPs, and runtime. “NR” indicates that the corresponding metric was not reported. Sensor-level latency or event rate is distinguished from application-level processing latency, and sensor-path power is distinguished from complete system power. Because the studies use different sensors, datasets, processors, illumination conditions, and evaluation protocols, values across rows should not be interpreted as a controlled head-to-head benchmark.

The matrix shows that no architecture has yet been evaluated comprehensively across all relevant system-level metrics. Dual-camera and optically aligned systems provide substantial gains in detection robustness and reconstruction quality, but numerical calibration residuals and complete system power are rarely reported. Pixel-level shared systems provide intrinsic event–frame correspondence and have demonstrated improved SLAM accuracy, although application latency and power are not consistently documented. Stacked systems provide the most complete sensor-level reporting, including resolution, dynamic range, power, and color or reconstruction quality, but computational cost remains algorithm-dependent. Homogeneous-pixel sensing preserves dense CIS sampling and quantitatively improves motion sharpness, while its approximately 1 ms temporal sampling and 845 mW power illustrate the cost of high-speed frame readout. Event-only reconstruction systems can achieve low application latency and reduced FLOPs, but reconstruction quality, downstream processing power, and static-scene performance remain strongly dependent on the algorithm.

## 6. Conclusions

Hybrid event–frame sensing provides a promising direction for next-generation vision systems by combining the complementary strengths of frame-based image sensing and event-based temporal sensing. Frame sensors provide dense spatial information, color fidelity, and compatibility with mature image-processing pipelines, whereas event sensors provide low-latency, sparse, and motion-sensitive information. By integrating these two sensing principles at the module, optical, pixel, wafer-stack, readout, or algorithmic level, hybrid event–frame systems can support a wide range of human-perceptual and machine-vision applications. This review classified representative hybrid event–frame sensing architectures and systems, including dual-camera event–frame systems, optically aligned event–frame systems, pixel-level shared hybrid image sensors, stacked CIS–DVS hybrid image sensors, homogeneous-pixel sensing systems, and event-only reconstruction systems. Each architecture offers different trade-offs in latency, spatial resolution, color fidelity, power consumption, and form factor. Therefore, the optimal hybrid event–frame architecture should be selected according to application requirements rather than by a single sensor metric. Among the reviewed architectures, stacked CIS–DVS sensors and homogeneous-pixel hybrid sensing systems are particularly attractive for compact and practical implementation. Stacked sensors provide high integration density and synchronized multimodal sensing, while homogeneous-pixel sensing systems preserve conventional CIS image quality and module compatibility. However, important challenges remain, including color-fidelity degradation caused by event-pixel insertion, demosaicing, calibration complexity, event-threshold mismatch, power-efficient fusion, thermal management, and benchmark standardization. Future hybrid event–frame sensing systems should be developed through sensor–algorithm–ISP–AI co-design. The pixel architecture, event-pixel ratio, readout circuit, demosaicing algorithm, event representation, fusion strategy, and edge-AI processor should be optimized together. Such co-design will be essential for achieving low latency, high image quality, compact form factor, and energy-efficient perception in real-world vision systems. In conclusion, hybrid event–frame sensing should not be viewed as a simple combination of RGB and event cameras. It represents a new sensing paradigm in which dense spatial/color information and sparse temporal information are jointly captured, processed, and interpreted. As sensor integration, computational imaging, and edge-AI technologies continue to advance, hybrid event–frame sensing is expected to become a key foundation for future human and machine vision systems.

## Figures and Tables

**Table 1:** Comparison of frame-based sensors, event-based sensors, and hybrid event–frame systems.

Sensor Type	Advantages	Limitations	Representative Applications
Frame-based RGB image sensor	High spatial resolution; dense texture and color information; human-interpretable images; mature ISP and computer-vision pipelines; compatibility with large RGB datasets.	Motion blur under long exposure; limited temporal resolution at low frame rate; redundant data output in static scenes; high bandwidth and power at high resolution or high frame rate; limited dynamic range for single-exposure imaging.	Mobile imaging, surveillance, human-viewable video, object recognition, scene understanding, automotive cameras, industrial inspection.
Event-based sensor	Very low latency; high temporal resolution; high dynamic range; sparse and activity-driven output; reduced redundant data; suitable for high-speed motion and low-power sensing.	No direct dense RGB or absolute intensity output; weak response to static scenes; event noise and background activity; dependence on bias and contrast thresholds; irregular asynchronous data require specialized algorithms.	High-speed motion analysis, optical flow, gesture recognition, robotics, visual odometry, SLAM, automotive perception, neuromorphic edge sensing.
Hybrid image sensor	Combines dense spatial/color or intensity information with fast temporal event information; improves motion robustness, latency, and energy efficiency; supports both human-perceptual imaging and machine vision.	Increased sensor and system complexity; spatial/temporal calibration issues in multi-sensor systems; increased per-pixel circuit complexity in pixel-level shared sensors; color-fidelity and demosaicing challenges in stacked sensors; more complex fusion algorithms and benchmarks.	Motion blur-free imaging, video deblurring, frame interpolation, SLAM, object/person detection, gesture recognition, AR/VR, robotics, automotive perception, and always-on AIoT sensing.

**Table 2:** Taxonomy of hybrid event–frame sensing architectures and systems.

Architecture Type	Integration Level	Advantages	Limitations	Suitable Applications
Dual camera	Module-level hybrid vision system	High flexibility; easy prototyping; independent sensor selection; suitable for dataset collection and algorithm evaluation.	Parallax; synchronization error; extrinsic calibration complexity; larger form factor; higher module-level power.	Robotics, autonomous driving datasets, object detection, SLAM, visual odometry, multimodal perception research.
Optically aligned	Module-level hybrid vision system	Reduced parallax; improved event–frame registration; suitable for dense fusion, deblurring, interpolation, and controlled experiments.	Light loss due to beam splitting; optical complexity; larger optical module; residual calibration still required.	Event-guided deblurring, frame interpolation, HDR/low-light imaging, image restoration, dataset acquisition.
Pixel-level shared	Physically integrated hybrid image sensor	Intrinsic spatial alignment between frame and event outputs; no external event–frame geometric calibration; compact single-sensor implementation; synchronized APS intensity frames and DVS events.	Increased per-pixel circuit complexity; reduced fill factor or larger pixel pitch; limited color capability in many DAVIS implementations; dual-mode readout complexity; less flexibility than dual-camera systems.	Visual odometry, SLAM, high-speed tracking, robotics, event-guided reconstruction, visual–inertial perception, and event–frame algorithm benchmarking.
Stacked	Physically integrated hybrid image sensor	High functional density; compact single-chip form factor; parallel CIS and DVS data paths; high event throughput; on-chip ISP/ESP possible.	High fabrication complexity; Cu–Cu bonding yield; inter-layer noise; thermal coupling; calibration and testing difficulty.	High-end mobile imaging, automotive perception, robotics, AR/VR, high-resolution hybrid sensing.
Homogeneous-pixel	Computationally hybrid vision system	Preserves regular CIS pixel layout; avoids event-pixel-induced static artifacts; compatible with small CIS pixel pitch and mature ISP pipelines.	Pseudo-events are discrete-time; latency limited by high-speed readout; not equivalent to true asynchronous DVS; higher internal readout bandwidth may be required.	Motion blur-free imaging, high-speed video reconstruction, mobile human-perceptual imaging, frame-domain event-like sensing.
Event-only algorithmic reconstruction	Computationally hybrid vision system	Event-only hardware; high temporal resolution; high dynamic range; frame-like grayscale output can be generated without a physical frame sensor.	Ill-posed intensity recovery; no direct color information; static scene ambiguity; dependence on learned priors and computation; possible hallucination or smoothing artifacts.	Event-camera visualization, high-speed grayscale video generation, robotics, event-based perception, downstream image-based algorithms.

**Table 3:** Illustrative RGB-to-DVS design hypotheses and the published MIPI 2024 feasibility point.

RGB-to-DVS Ratio	Event-Pixel Occupancy	Evidence Level	Design Interpretation
31:1	3.125%	Preliminary hypothesis	Low event-density, image-quality-oriented design point; not experimentally validated
15:1	6.25%	Preliminary hypothesis	Intermediate trade-off hypothesis; not established as a practical baseline
7:1	12.5%	Published MIPI 2024 evidence	Feasible under the specific challenge pattern, dataset, algorithms, and PSNR/SSIM metrics

**Table 4:** Comparison of representative CIS–DVS hybrid image sensing technologies in terms of pixel ratio, pixel pitch, and power consumption.

Specifications	A [55,56]	B [23]	C [36]
Architecture	Stacked	Stacked	Homogeneous-pixel
CIS resolution	4096 × 3680 (15 Mp)	35.6 Mp	4032 × 3024 (12 Mp)
Pixel ratio	15:1	3:1	N/A
CIS pixel pitch (μm)	2.2	1.22	1.8
DVS pixel pitch (μm)	8.8	4.88	1.8
Power Consumption (mW)	64 (DVS-only)	525	845
Remarks	High-throughput DVS readout up to 4.6 GEvents/s with in-pixel TDC and on-chip ISP/ESP functions.	High-resolution RGB CIS combined with event pixels; adaptive event-sparsity control supports up to 10,000 event frames/s.	Pseudo-DVS is generated from high-speed CIS frame differencing; no static bad pixels are introduced by dedicated DVS pixels.

**Table 5:** Representative measured application-level outcomes of hybrid event–frame sensing architectures.

Architecture	Applications	Latency or Rate	Performance	Dynamic Range/Power	Calibration/ Computational Cost
Dual-camera hybrid vision system [196]	Roadside RGB–event object detection using the TUMTraf Event dataset	NR	Event–frame fusion improved detection performance by up to 9% mAP during daytime and 13% mAP at night relative to RGB-only detection	NR	Targetless extrinsic calibration for multiple moving objects; numerical residual calibration error NR
Dual-camera CIS–DVS system [208]	Post-capture motion-deblurred image restoration	NR	PSNR: 18.52 → 38.72 dB; SSIM: 0.683 → 0.911; normalized MTF50 ratio: 0.39 → 0.99	NR	Spatial, temporal, resolution, and disparity compensation; computational cost NR
Optically aligned event–frame system [200]	Beam-splitter-based video frame interpolation using Time Lens++	NR	Reconstruction improved by up to 0.2 dB PSNR and 15% LPIPS; dataset contained more than 100 scenes	NR	Beam-splitter-based spatial alignment; numerical residual calibration error NR
Pixel-level shared hybrid sensor [11]	Event–frame–IMU visual SLAM using a DAVIS-type sensor	NR	Accuracy improved by 130% over an event-only pipeline and 85% over a frame-only visual–inertial pipeline	NR	Intrinsically co-located frame and event measurements; IMU synchronization required
Stacked CIS–DVS sensor [23]	High-resolution RGB and event acquisition	Up to 10,000 event frames/s for the 35.6-Mpixel sensor; up to 4.6 GEvents/s for the three-wafer-stacked sensor	35.6-Mpixel RGB with 4.88 μm event pixels; three-wafer-stacked implementation with 15-Mpixel CIS and 1-Mpixel EVS	67.8 dB/ 525 mW	NR
Stacked/inserted-pixel HybridEVS [160,161,162,163,164]	Quad-Bayer HybridEVS demosaicing	NR	Best MIPI 2024 result: 44.8464 dB PSNR and 0.9854 SSIM for the approximately 7:1 RGB-to-DVS pattern	NR	NR
Pixel-aligned RGB–event ISP system [165]	Image enhancement	Processing time: 10 ms	Overall average PSNR of 32.47 dB	NR	InvertISP excels in computational efficiency with 1.41 GFLOPs
Homogeneous-pixel pseudo-DVS system [36]	Motion-blur-free hybrid image sensing	1440 fps pseudo-DVS output; approximately 1 ms event-like latency	Normalized MTF50 ratio improved from approximately 0.4 to 0.8 after motion compensation	Approximately 845 mW for the demonstrated high-speed implementation	NR
Event-only computational reconstruction [18,73]	Events-to-video reconstruction using E2VID	Reconstructed-frame interval configurable; complete runtime depends on implementation	Reported to outperform preceding reconstruction methods by more than 20% in image quality	NR	NR
Event-only machine vision [209]	Human detection, pose estimation, and hand-posture recognition for edge AI	Human detection: 15 ms; pose estimation: 6 ms; hand posture: 14.31 ms	Human-detection computation: 5.8 G → 81 M FLOPs; pose mAP: 0.95 → 0.94; hand-posture recall: 99.19%, FAR: 0.0926%	NR	Pose-model size: 127 → 19 MB; detection speed-up greater than 11×
Event-only low-latency machine vision [210]	Person detection, gesture recognition, and SLAM on mobile processors	Person detection: 92 ms; gesture recognition: 20 ms; SLAM: 15.9 ms	Task-specific accuracy and robustness metrics reported separately in the source	NR	Exynos 7570, Exynos 5422, and Snapdragon 845; task-specific event representations

## Data Availability

3rd Party Data. Restrictions apply to the availability of these data.

## Abbreviations

The following abbreviations are used in this manuscript:

ADC	Analog-to-Digital Converter
AER	Address-Event Representation
AI	Artificial Intelligence
AIoT	Artificial Intelligence of Things
APS	Active Pixel Sensor
AR	Augmented Reality
VR	Virtual Reality
BSI	Backside Illumination
CDS	Correlated Double Sampling
CFA	Color Filter Array
CIS	CMOS Image Sensor
CNN	Convolutional Neural Network
CMOS	Complementary Metal-Oxide Semiconductor
CV	Computer Vision
DNN	Deep Neural Network
DR	Dynamic Range
DVS	Dynamic Vision Sensor
EVS	Event Vision Sensor
FAR	False Acceptance Rate
FD	Floating Diffusion
FLOPs	Floating-Point Operations
FPN	Fixed-Pattern Noise
HDR	High Dynamic Range
IMU	Inertial Measurement Unit
ISP	Image Signal Processing
LPIPS	Learned Perceptual Image Patch Similarity
mAP	mean Average Precision
MIPI	Mobile Industry Processor Interface
MTF	Modulation Transfer Function
PPD	Pinned Photodiode
PSNR	Peak Signal-to-Noise Ratio
QCFA	Quad Color Filter Array
RGB	Red, Green, and Blue
ROI	Region of Interest
RTK GPS	Real-Time Kinematic Global Positioning System
SLAM	Simultaneous Localization and Mapping
SNR	Signal-to-Noise Ratio
SSIM	Structural Similarity Index Measure
UAV	Unmanned Aerial Vehicle
